# Advances in acute respiratory distress syndrome: focusing on heterogeneity, pathophysiology, and therapeutic strategies

**DOI:** 10.1038/s41392-025-02127-9

**Published:** 2025-03-07

**Authors:** Wen Ma, Songling Tang, Peng Yao, Tingyuan Zhou, Qingsheng Niu, Peng Liu, Shiyuan Tang, Yao Chen, Lu Gan, Yu Cao

**Affiliations:** 1 Department of Emergency Medicine, Institute of Disaster Medicine and Institute of Emergency Medicine, West China Hospital, Sichuan University, Chengdu, China; 2 Institute for Disaster Management and Reconstruction, Sichuan University-The Hong Kong Polytechnic University, Chengdu, China

**Keywords:** Respiratory tract diseases, Cell biology

## Abstract

In recent years, the incidence of acute respiratory distress syndrome (ARDS) has been gradually increasing. Despite advances in supportive care, ARDS remains a significant cause of morbidity and mortality in critically ill patients. ARDS is characterized by acute hypoxaemic respiratory failure with diffuse pulmonary inflammation and bilateral edema due to excessive alveolocapillary permeability in patients with non-cardiogenic pulmonary diseases. Over the past seven decades, our understanding of the pathology and clinical characteristics of ARDS has evolved significantly, yet it remains an area of active research and discovery. ARDS is highly heterogeneous, including diverse pathological causes, clinical presentations, and treatment responses, presenting a significant challenge for clinicians and researchers. In this review, we comprehensively discuss the latest advancements in ARDS research, focusing on its heterogeneity, pathophysiological mechanisms, and emerging therapeutic approaches, such as cellular therapy, immunotherapy, and targeted therapy. Moreover, we also examine the pathological characteristics of COVID-19-related ARDS and discuss the corresponding therapeutic approaches. In the face of challenges posed by ARDS heterogeneity, recent advancements offer hope for improved patient outcomes. Further research is essential to translate these findings into effective clinical interventions and personalized treatment approaches for ARDS, ultimately leading to better outcomes for patients suffering from ARDS.

## Introduction

Acute respiratory distress syndrome (ARDS) has emerged as a critical and complex medical issue in recent years. It is characterized by acute hypoxaemic respiratory failure, accompanied by diffuse pulmonary inflammation and bilateral edema, which stem from excessive alveolocapillary permeability in patients suffering from non-cardiogenic pulmonary diseases. The Berlin definition, currently regarded as the gold standard, dominates the diagnostic landscape for ARDS. However, no foolproof method can definitively confirm or rule out this diagnosis. Compounding this diagnostic conundrum is the remarkable heterogeneity that characterizes ARDS. The underlying pathological causes, the way they present clinically, and how patients respond to treatment can vary dramatically from one individual to another. This variability presents a significant challenge to both clinicians working on the front lines and researchers dedicated to finding solutions.

Recent advances in research have yielded significant progress, particularly in the area of cell therapy. Mesenchymal stromal cells (MSCs), for example, have emerged as a promising therapeutic option due to their unique immunomodulatory and regenerative properties. These cells have shown potential in modulating the immune response and hastening tissue repair in the lungs. However, several challenges remain plentiful. One of the key obstacles is selecting the most suitable cell source. Options such as bone marrow-derived, adipose-derived, or umbilical cord-derived MSCs each have their pros and cons. Deciding on the optimal delivery method, whether it be intravenous injection for systemic effect, inhalation to target the lungs directly, or direct injection into the lung tissue for more precise targeting, is a topic of intense debate. Moreover, pinpointing the exact timing of intervention to achieve maximum therapeutic benefit is equally contentious. In the realm of targeted therapy, scientists have been painstakingly investigating specific molecular pathways implicated in ARDS pathogenesis. However, issues like off-target effects that could lead to unwanted side effects in other organs, potential drug resistance over time, and the need for comprehensive long-term safety evaluations have come to the fore. Personalized therapy is gaining momentum, intending to tailor treatment strategies based on individual patient profiles. However, the task of collecting and analyzing vast amounts of patient data to create an accurate and comprehensive profile, while integrating numerous factors into an effective treatment plan, remains a monumental challenge.

This review is wholeheartedly committed to bridging the knowledge gaps by conducting an exhaustive exploration of the current state of the art. Its primary objective is to succinctly analyze the heterogeneity, and pathophysiology of ARDS, with a particular focus on cell therapy, targeted therapy, and personalized therapy. By doing so, we aim to provide valuable insights and ultimately lead to improved patient outcomes.

## Historical perspective on the definition of ARDS

**Fig. 1:**
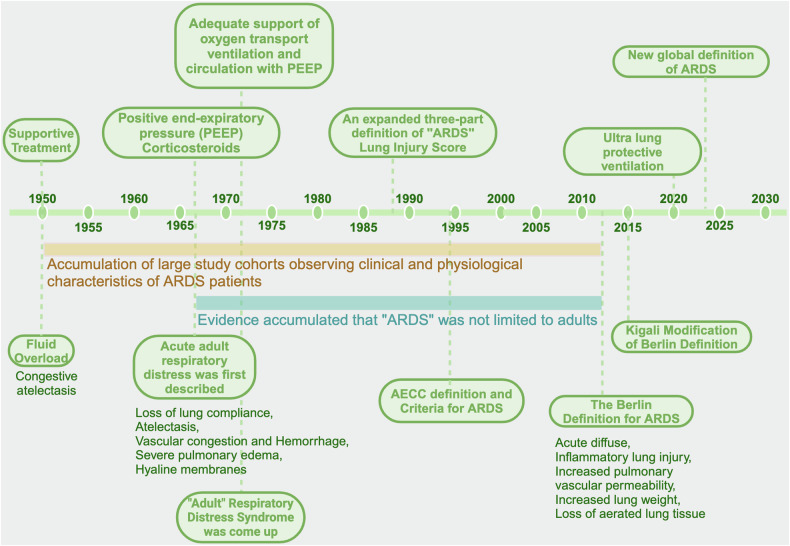
**70 years history of ARDS**. Over the past 70 years, as research on ARDS has deepened, the definition of ARDS has gradually evolved, from “fluid overload” to the “Berlin definition”. This diagram shows important nodes in the evolution of ARDS definitions

Before the 1960s, fluid overload was deemed the sole causative agent of congestive atelectasis according to clinical inspections and microscopic and macroscopic postmortem findings.^1^ Ashbaugh and colleagues initially described acute respiratory distress in adults with pathologic examination results, including loss of lung compliance, atelectasis, vascular congestion and hemorrhage, severe pulmonary edema, and hyaline membranes.^2^ Moreover, treatment with positive end-expiratory pressure (PEEP) and corticosteroids was found to be helpful. In 1971, “adult” respiratory distress syndrome was first reported as the abbreviation of the principles of management, including adequate support for oxygen transport, ventilation, and circulation using volume respirators with PEEP.^3^ Murry and coworkers proposed an expanded three-part definition of ARDS accompanied by a “lung injury score” that detailed the disease state, severity, and underlying cause or associated condition.^4^ Additional evidence later revealed that ARDS is not limited to adults but also occurs in pediatric patients.^5,6^ Therefore, the term “adult” was no longer appropriate as part of the definition of ARDS. In 1994, the first American-European Consensus Conference (AECC) issued criteria for acute lung injury (ALI) and ARDS, which included timing (acute onset), oxygenation, chest radiography, and elevated pulmonary capillary pressure, with “acute” used as part of the new definition of ARDS.^7^ However, the lack of standard criteria for diagnosing acute onset, the sensitivity of the oxygenation index to different ventilation settings, the reliability of chest radiography criteria, and the difficulty of distinguishing hydrostatic edema made it difficult for physicians to diagnose ARDS. After the publication of the clinical and physiological characteristics of ARDS patients in large study cohorts in 2012, the Berlin definition was proposed and stated that “ARDS is a type of acute diffuse, inflammatory lung injury, leading to increased pulmonary vascular permeability, increased lung weight, and loss of aerated lung tissue.” The Berlin definition provides more precise criteria for diagnosing ARDS, including timing, radiographic evidence, edema origin, and severity based on the oxygenation index and PEEP levels.^8^ The accurate and evidenced-based Berlin definition achieved a better ability to predict mortality.^8^ In 2015, the Kigali modification, which proposed a SpO2/FIO2 ratio ≤ 315 irrespective of PEEP as an alternative criterion, provided insights for diagnosing ARDS in resource-constrained regions where mechanical ventilators, arterial blood analysis, and chest radiography are unavailable.^9^ After over 70 years of struggling to treat ARDS, supportive care remains the main treatment approach, and few drugs have been proven effective for all patients.^10,11^ The etiology, physiology and microbiology of ARDS are highly heterogeneous,^10^ so personalized medicine approaches for patients with different phenotypes might be a goal of future treatment. The important stages in the evolution of ARDS definitions are shown in Fig. 1.

However, as our understanding of ARDS deepens, we should also be aware of some limitations in the definition of ARDS. The SpO2/FiO2 ratio has been clinically validated for diagnosing and stratifying ARDS patients, but it may be less accurate in poor perfusion states, above 97% oxygen saturation, and in patients with darker skin pigmentation.^12^ Bilateral opacities on chest X-rays were part of the Berlin Definition for ARDS, but they lack interobserver reliability. The radiographic diagnostic accuracy and agreement among raters were poor with the Berlin definition.^13^ The “Radiographic Assessment of Lung Edema” (RALE) score was introduced to assess the extent and density of alveolar opacities on chest X-rays in ARDS patients, showing preferable diagnostic accuracy.^14^ Encouragingly, recent research indicated that an AI-based model utilizing chest X-rays and incorporating specific LUS criteria^15,16^ could enhance the specificity and sensitivity of ARDS definitions.^17^

## Epidemiology

In recent years, the incidence of ARDS has shown a gradual upward trend,^18–20^ which is related to the introduction of the Berlin definition and an improved ability to identify ARDS patients.^21^ The incidence of ARDS is age dependent, increasing from 16/100,000 person-years for individuals 15–19 years of age to 306/100,000 person-years for individuals 75–84 years of age.^18^ In addition, the incidence of ARDS is also gender dependent, and men (62%) are more likely to develop ARDS than women (38%).^22^ The incidence of ARDS among ICU patients was investigated in a large-scale study; 10% of ICU patients met the criteria for ARDS, and a quarter of all critically ill patients who required mechanical ventilation developed ARDS.^23^ Despite advances in supportive care, ARDS remains a significant cause of morbidity and mortality in critically ill patients, with high mortality rates of 35% (for mild cases), 40% (for moderate cases), and 45% (for severe cases).^8,23–25^ However, there is no significant difference in overall hospital mortality between men (40.2%) and women (40.2%), although women have been reported to have higher mortality in patients with severe ARDS.^22^ In China, comprehensive studies of the prevalence, mortality, and risk factors of ALI/ARDS are lacking. However, a few regional studies have suggested that the epidemiology of ARDS in China is similar to that in Europe and the United States. Of 1814 patients in 20 ICUs in 9 provinces in China, 147 (8.1%) ICU patients met the criteria for ARDS, with an in-hospital mortality rate of 34.0%.^26^ However, in another multicentre prospective longitudinal study, the incidence of ARDS was low; of 18,793 ICU patients, only 3.6% met the criteria outlined in the Berlin definition of ARDS, with an in-hospital mortality rate of 244 (46.3%).^27^ Moreover, the epidemiology of COVID-19-related ARDS (CARDS) in China has varied among different cohort studies. According to several small sample studies, the probability that a COVID-19 patient will suffer from ARDS is 40–65%, with a mortality rate ranging from 50–75%.^28,29^ However, a large study including 1875 COVID-19 patients suggested that 19.3% of COVID-19 patients will develop CARDS, which is similar to other reported data.^30^ The reason for this difference may be related to the sample size or the inclusion and exclusion criteria.

Pneumonia is the most common cause of ARDS, followed by extrapulmonary sepsis, aspiration, and trauma.^18,23,31^ Notably, some viruses that cause pneumonia are more likely to cause ARDS, including SARS-CoV (2003), H1N1 influenza (2009), MERS-CoV (2012), and most notably SARS-CoV-2 (2019), which led to the COVID-19 pandemic. Of those hospitalized for COVID-19, 15–30% typically develop CARDS.^32,33^ Substantial evidence has shown that smoking cigarettes^34,35^ and chronic consumption of large amounts of alcohol^36^ increase the risk of developing ARDS. Additionally, blood product transfusion^37^ and e-cigarette or vaping product use-associated lung injury (EVALI) were identified as risk factors for ARDS in several trials.^38–40^ Notably, long-term exposure to air pollutants, especially ozone, has also been reported to be a potential modifiable environmental risk factor for ARDS.^41,42^

## Pathogenesis of ARDS

**Fig. 2:**
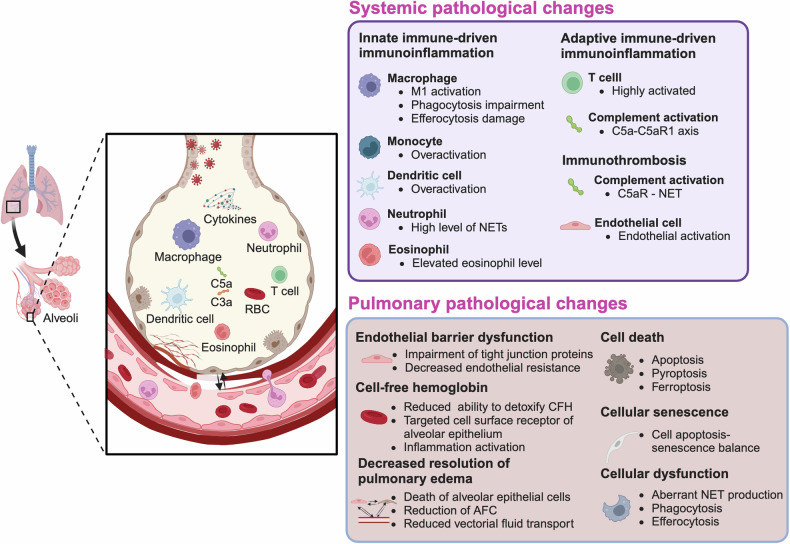
**The pathological changes in ARDS**. Acute respiratory distress syndrome affects not only pulmonary tissue but also extra-pulmonary tissues. Systemic pathological changes, such as immunoinflammation and immunothrombosis, occur throughout the body, and various cells are involved in these pathological changes including macrophage, monocyte, dendritic cell, neutrophil, eosinophil, T cell and endothelial cell. These changes are believed to contribute to cellular abnormalities within the pulmonary tissue, ultimately leading to damage to the alveolar-capillary barrier in ARDS. A variety of pulmonary pathological changes have been observed in the damaged lung tissue, including endothelial barrier dysfunction, the presence of cell-free hemoglobin, reduced resolution of pulmonary edema, cell death, cellular senescence, and cellular dysfunction. *M1: Proinflammatory phenotype of macrophages; NET: neutrophil extracellular trap; CFH: cell-free hemoglobin; AFC: alveolar fluid clearance

The pathophysiology of ARDS is complex, and the mechanism includes the activation and dysregulation of multiple overlapping and interacting pathways associated with injury, inflammation, and coagulation, both in the lungs and systemically; this process involves a variety of cells.^43^ The pathological changes that occur in ARDS include alveolar epithelial injury, pulmonary endothelial injury, pulmonary macrophage injury, and pulmonary fibroblast injury, which are all observed in animal models of ALI/ARDS. Oxidative stress, inflammation, apoptosis, and barrier breakdown are observed in alveolar epithelial cells and pulmonary endothelial cells in mice with ALI,^44–47^ with increased levels of alveolar injury markers and endothelial injury markers.^48,49^ We provide a detailed summary of the pathological changes and potential mechanisms of ARDS below, shown in Fig. 2.

### Systemic pathological changes and underlying mechanisms

ARDS is a syndrome that can impact pulmonary tissue as well as extrapulmonary tissues. Several systemic pathological changes, such as immune dysfunction, inflammatory responses, and abnormal coagulation, have been reported to occur during ARDS. Herein, we focus on the interactions between immune dysfunction and inflammation and the interactions between coagulation disorders and inflammation in the context of lung thrombosis, which are classified as immunoinflammation and immunothrombosis, respectively.

#### Immunoinflammation

Immunoinflammation, the initial immunological response that leads to inflammation and initiates a vicious cycle of immune responses and inflammation,^50,51^ is closely associated with the molecular pathogenesis of ARDS. In ARDS caused by infection, immune responses, including innate and adaptive immune responses, are first activated by pathogens. However, in noninfective ARDS, alveolar epithelial cell injury, pulmonary endothelial activation, and alveolar macrophage responses can recruit neutrophils and natural killer (NK) cells as well as monocytes, such as macrophages and dendritic cells, from the circulation, thus initiating the immune response during ARDS.^52,53^ An active immune response ultimately leads to immune disorders in ARDS patients, further promoting the inflammatory response through similar effector cells in both immune and inflammatory aetiologies. The activation of the adaptive immune system in ARDS involves T lymphocytes, B lymphocytes, antigen-presenting cells (APCs), and several influential factors, such as complement. We have summarized the cells and molecules involved in the above information in Table 1.^54–69^

**Table 1:** Cells involved in immunoinflammation of ARDS

	Cell type	Pathological process	Pathological changes	Outcome	Ref
**Innate immune-driven immunoinflammation**	Macrophage	Polarized into pro-inflammatory phenotypes	Elevated levels of SP-D and anti-spike antibodies	Macrophage dysfunction	^54,55^
		Phagocytosis is impaired	Increased level of SIRP-alpha		^56^
		Efferocytosis is impaired	Elevated levels of HMGB1; VEGF-C/VEGFR-3 signaling pathway		^57,58^
	Monocyte	Aberrant activation	Strong monocyte/DC activation was observed with increasing levels of typical inflammatory markers	Additional innate immune disorder and severe inflammation	^59^
	DC				
	Neutrophils	Produce high level of NETs	Enhanced vascular damage	Severe cytokine storm	^60–62^
	Eosinophils	Elevated eosinophil level	Quick response to pulmonary injury	Inflammation	^63^
**Adaptive immune-driven immunoinflammation**	T cells	Overproduction of T lymphocytes	Abundant T cells are detected in the BALF	Hyperimmunoinflammation	^64,65^
		Activation of unconventional T cells	Highly activated in the airways of patients with CARDS		^66^
		Imbalance between effector T lymphocytes and the capacity to present antigens in APCs	Enhanced abundance of CD8 + T cells with decreased MHC-II expression in APCs		^67^
	C5a	Recruit neutrophils and monocytes from the circulation to the lung	C5a-C5aR1 axis is activated and promote immune dysfunction and pulmonary inflammation	Complement-dependent immunoinflammation	^68^
	C3a	Complement activation induces excessive T cell cytotoxicity	Complement activation creates an inflammatory milieu that drives differentiation of T cells with high immunopathogenic potential		^69^

Several cells are involved in the immune response of ARDS, which in turn triggers a vicious cycle of immune response and inflammation

*HMGB1 high mobility group box 1, *DC* dendritic cell, *NETs* neutrophil extracellular traps, *MHC* major histocompatibility complex, *APCs* antigen-presenting cells

Several key signaling pathways are involved in immunoinflammation during ARDS, including macrophages and endothelial cells. IL33-STAT3-MMP2/9 is reported to play an important role in macrophage polarization from anti-inflammatory phenotype (M2) to proinflammatory phenotype (M1), thus inducing lipopolysaccharide (LPS)-induced ALI and pulmonary inflammation.^70^ Macrophage polarization further leads to endothelial injury. Extracellular nicotinamide phosphoribosyltransferase (eNAMPT) from endothelial cells is responsible for endothelial inflammation and the subsequent disruption in the endothelial barrier, which depends on the TLR4 inflammatory pathway.^71^ Both macrophage and endothelial changes contribute to immunoinflammation in ARDS.

#### Immunothrombosis

Immunothrombosis, the interaction between the coagulation system and the innate immune system after infection, is an emerging pathogenic mechanism in ARDS, especially in CARDS. Neutrophils, macrophages, and other effectors are involved in the innate immune response, and platelets are the primary cells involved in immunothrombosis and complement signaling.^72^ The complement system participates in the immunothrombosis process in CARDS. Complement activation has been reported to be associated with enhanced thrombotic activity, and blockade of C5aR1 can alleviate platelet-mediated thrombogenicity in a neutrophil extracellular trap (NET)-dependent manner in ARDS.^73^ Increased NET formation is associated with microthrombus and platelet accumulation in the pulmonary circulation, indicating that NETs promote immunothrombosis in ARDS.^72,74^ Additionally, endothelial activation plays a pivotal role in immunothrombosis and aberrant coagulation since elevated levels of endothelium-derived glycoproteins have been detected in ARDS patients with blood type A and are associated with an increased risk of disseminated intravascular coagulation.^75,76^

There are also several key signaling pathways involved in the immunothrombosis during ARDS, especially in neutrophils. Formation of NETs is essential in immunothrombosis, and it relies on the CLEC5A-TLR2 activation in CARDS as extremely low level of NETs is detected in neutrophils with both CLEC5A and TLR2 ablation.^77^ While in bacterial infection-induced ARDS, CXCL2-CXCR2 signaling activation of neutrophils is more significant,^78^ because CXCL2 is more sensitive in response to molecules of bacterial origin.

### Pulmonary pathological changes and underlying mechanisms

Due to systemic and pulmonary immunoinflammatory and immunothrombosis during ARDS, as well as subsequent disruption of the alveolar-capillary barrier, various cellular pathological changes, including various types of cell death, cellular senescence, and cellular dysfunction, have been demonstrated to occur in damaged pulmonary tissue. The potential underlying mechanism involves disruption of intracellular functions, such as excessive ROS accumulation, endoplasmic dysfunction, and mitochondrial dysfunction.

#### Endothelial barrier dysfunction

The impairment or degeneration of tight junction proteins is thought to accelerate endothelial barrier disruption and play a vital role in hyperpermeability during ARDS. Plasma from severe COVID-19 ARDS patients could impair the endothelial barrier integrity of primary human pulmonary microvascular endothelial cells in vitro, as indicated by decreased endothelial resistance measured by electrical cell impedance sensing (ECIS), transendothelial electrical resistance (TEER) and loss of occludin.^79^ Moreover, reductions in pulmonary ZO-1 and occludin levels were also observed in sepsis-related ARDS patients.^47^ In the endothelial barrier injury associated with ARDS, several signaling pathways have been identified as key regulators, including METTL3-mediated N6-methyladenosine modification of tripartite motif-containing (Trim)59 mRNA,^80^ NOX4 activation of CaMKII/ERK1/2/MLCK and Akt-FoxO1/3a signaling pathways,^81^ as well as CREB-mediated transcription of VE-cadherin.^82^ Although animal experiments targeting these signaling pathways have demonstrated efficacy, further research is needed to confirm their feasibility.

#### Cell-free hemoglobin

After the pulmonary capillary endothelium was destroyed, proteinaceous fluid and white blood cells flew into the alveolus, causing diffuse lung inflammation and coagulation.^83^ Red blood cells (RBCs) also crossed the pulmonary capillary endothelium and could be found in the alveoli of patients with ARDS.^84,85^ Lysis of RBCs within the intravascular and alveolar spaces results in the release of cell-free hemoglobin (CFH),^86^ which was harmful.^85^

The mechanism of CFH injury in acute respiratory distress syndrome may be specifically mediated through targeted cell surface receptor binding on the alveolar epithelium, as an in vitro study demonstrated that supplementation with antioxidants or iron chelators did not alter the effect of methemoglobin.^87^ Additionally, hemoglobin increased leukocyte-endothelial adhesion and activated lung microvascular endothelial cells through TLR4 signaling under inflammatory and hemolytic conditions.^88^ Similarly, a recent study of Schaaf KR reported that CFH was elevated in the airspace of most patients with ARDS and caused severe inflammation, and TLR4 on alveolar macrophages mediated the CFH-induced lung inflammation.^89^ These data suggest that TLR4 may be the key to targeting CFH in ARDS treatment.

CFH could be detected in the plasma of 80% of patients with sepsis,^90^ which was associated with mortality in patients with ARDS.^91^ This data has been used to argue the design of a clinical trial aimed at investigating acetaminophen’s effectiveness as a treatment for ARDS.^92^

#### Decreased resolution of pulmonary edema

Most patients with ARDS had impaired ability to clear pulmonary edema, which was associated with a higher mortality rate.^93^ Several mechanisms influence the resolution of alveolar edema in patients with ARDS, with the death of alveolar epithelial cells being the primary mechanism.^94^

Sodium enters through apical channels, especially the epithelial sodium channel (ENaC), and is then expelled into the lung interstitium by the Na/K-ATPase situated on the basolateral side. This process generates a localized osmotic gradient that facilitates the reabsorption of the water content from the edema fluid within the airspaces of the lungs.^94^ Moderate hypoxemia reduced apical sodium uptake through transcriptional effects and impaired ENaC transport, resulting 50% reduction of alveolar fluid clearance (AFC).^95^ In addition, cytokines in the airspace, including IL-1β, IL-8, and TGF-β, reduced vectorial fluid transport in alveolar epithelial cells by decreasing the expression and function of Na/K-ATPase and ENaC.^96–100^ There was also evidence that alveolar epithelial cell injury and dysfunction might be partially caused by mitochondrial damage leading to low intracellular ATP levels.^101^ Influenza virus infection could impair the function of ENaC, while bacterial and viral products could damage alveolar epithelial cells directly or indirectly.^102–104^ Therefore, multiple factors may lead to decreased resolution of pulmonary edema, revealing the complexity of improving AFC.

#### Cellular pathological changes

**Fig. 3:**
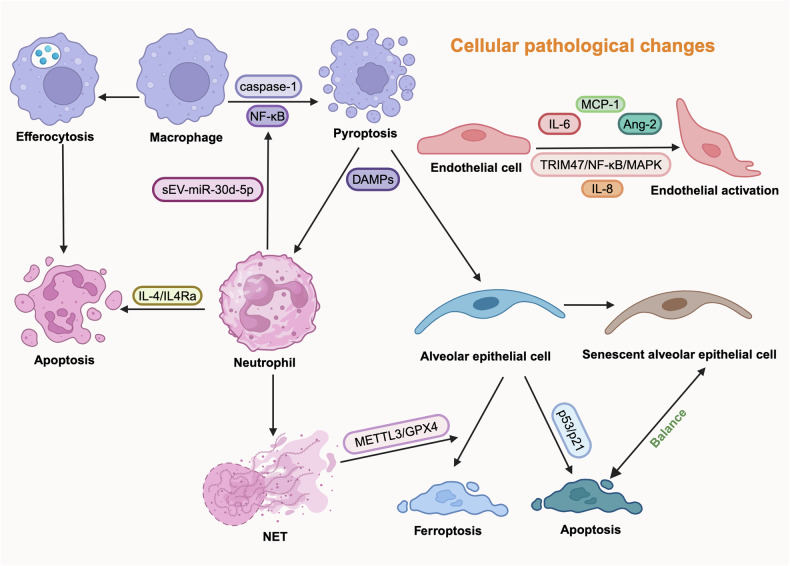
**Cellular pathological changes in pulmonary tissue**. In the pathological process of ARDS, neutrophils, macrophages, alveolar epithelial cells, endothelial cells, etc., interact with each other through various cytokines, leading to pathological changes including efferocytosis, NET formation, senescence, apoptosis, pyroptosis, ferroptosis, etc. *DAMPS damage-associated molecular patterns, MCP monocyte chemoattractant protein, MAPK mitogen-activated protein kinase, IL interleukin, METTL methyltransferase, GPX glutathione-peroxidase, NET neutrophil extracellular trap

In the pathophysiology of ARDS, macrophages, neutrophils, alveolar epithelial cells, endothelial cells, etc., influence the pathological progression of ARDS through various mechanisms, mainly including cell death, cellular senescence, and cellular dysfunction, shown in Fig. 3.^60,105–115^

## Classifications and phenotypes

ARDS exhibits clinical heterogeneity. ARDS can be divided into subphenotypes based on clinical features, causes of lung injury, effective biomarkers, or clinical and biological variables. This heterogeneity may explain the lack of benefit observed in most randomized controlled trials (RCTs) evaluating various treatment strategies.^116^ One study reported that the ARDS subphenotype was stable within the first three days of enrollment, indicating that subphenotypic identification is feasible in clinical trials.^117^ Accurate subphenotypic classification of ARDS will improve clinical outcomes.

### Clinical subphenotypes

Latent class models are applied to identify subphenotypes. Based on the clinical and biological data of two ARDS RCTs, Calfee CS et al. identified two ARDS subphenotypes (hyperinflammatory and hypoinflammatory).^118^ Compared to the hypoinflammatory subphenotype, the hyperinflammatory subphenotype was characterized by higher plasma levels of inflammatory biomarkers, a greater incidence of vasopressor use, lower serum bicarbonate levels, and a greater incidence of sepsis, with higher mortality and fewer ventilator-free days (VFDs) and organ failure-free days in both the ALVEOLI and ARMA cohorts.^118^ Data from several large-scale RCT studies indicates that the proportion of the hyperinflammatory phenotype is approximately 30–40%^118–121^ Multiple subsequent studies have demonstrated the existence of hyper and hypoinflammatory subphenotypes in different ways.^119–121^

A cluster analysis study that included 3875 ARDS patients identified three additional clinical subphenotypes of ARDS. Subphenotype I (40%) was associated with fewer abnormal laboratory values and less organ failure, with the lowest in-hospital mortality rate and the most VFDs and ICU-free days (IFDs), which was similar to the findings reported for the hypoinflammatory subphenotype. A higher white blood cell (WBC) count, higher temperature, a higher heart rate, a higher respiratory rate (RR), lower systolic blood pressure (SBP), and younger age characterized subphenotype II (32%), which was similar to the hyperinflammatory phenotype. Subphenotype III (28%) was characterized by older age, elevated serum creatinine and blood urea nitrogen (BUN) levels, and lower serum bicarbonate levels, with the least VFDs and IFDs, and the highest mortality rate, which was correlated with organ dysfunction, older age, and acidosis. Furthermore, these results were verified in three RCTs (ALVEOLI, FACTT, and SAILS trials),^122–124^ with a significant interaction between the three subphenotypes and treatment strategies in the ALVEOLI and FACTT trials. However, no apparent heterogeneity in treatment responsiveness was observed in the SAILS trial,^125^ suggesting that the treatment approach used may be effective across all subphenotypes of ARDS, or that the subphenotypes identified in the ALVEOLI and FACTT trials may not be as relevant or distinct in the SAILS population. It highlights the importance of considering the specific context and population of each trial when interpreting and generalizing research findings. These findings enhance the understanding of ARDS clinical subphenotypes and could be beneficial for the development of phenotype-specific treatment approaches.

### Rapidly improving ARDS subphenotypes

The LUNG SAFE study,^23^ a worldwide analysis of current ARDS epidemiology, found that nearly one-sixth of patients meeting the Berlin definition no longer met these criteria after 24 hours. Prior research suggested that using standardized ventilator settings could improve the Pao2 to Fio2 ratio (Pao2:Fio2) in some ARDS patients, leading to a Pao2:Fio2 ≤ 300 after 24 hours.^126–129^ This subphenotype was named rapidly improving ARDS (riARDS). Using data from the large ARDSNet clinical trial population, a secondary analysis of 4361 unique patients in randomized controlled trials indicated that 458 (10.5%) no longer met ARDS criteria on the first study day after enrollment, with better outcomes than ARDS lasting longer than one day.^130^ Despite the unclear underlying pathobiology, riARDS, typically defined by early extubation, is widely recognized as an increasingly prevalent subphenotype. However, by including data from 280 COVID-19 patients who received invasive mechanical ventilation during the second pandemic wave in three ICUs, it was found that riARDS was only present in 4% of patients and was linked to a 55% mortality rate.^131^ It seemed that riARDS was not common among COVID-19 patients and was not associated with any survival benefit, in contrast to previous reports for non-COVID-19-related ARDS. These findings might help us better understand the trajectory of ARDS and its relationship with prognosis in COVID-19 patients.

### Longitudinal phenotypes

ARDS is a dynamic process; most previous studies have captured only cross-sectional data, and longitudinal studies are relatively scarce. Recently, a study attempted to identify longitudinal phenotypes of ARDS and explore the dynamic changes in ARDS phenotypes.^132^ Chen H et al. identified a three-class model with different severities of pulmonary mechanics, organ dysfunction, chest CT features, and outcomes. Class 1 (66.1%) had fewer abnormal laboratory values and less organ dysfunction. Class 2 (16.9%), characterized by the highest minute ventilation, driving pressure, mechanical power (MP), and ventilatory ratio, and the lowest PaO_2_/FiO_2_, was called the pulmonary mechanical dysfunction phenotype. Class 3 (17%) was characterized by the highest creatinine and lactate levels, the lowest bicarbonate level and mean arterial pressure (MAP), and a greater proportion of patients who received vasopressors and was termed the extrapulmonary dysfunction phenotype. The authors also observed a significant interaction effect between phenotypes and the PEEP strategy when evaluating 60-day mortality. Significantly, most patients exhibited phenotypic changes at least once during the first four days of invasive ventilation, which shows the significance of this longitudinal phenotype study.

### COVID-19-related phenotypes

Since the COVID-19 pandemic, studies have attempted to determine the prevalence of previously described ARDS phenotypes in patients with CARDS. A preliminary analysis by Sinha P suggested that the hyperinflammatory phenotype of ARDS was less prevalent in COVID-19 patients than in patients in previous ARDS cohorts, which challenges the theory that a cytokine storm is involved in CARDS.^133^ However, two subphenotypes of CARDS were proposed, and class 2 showed increased expression markers of coagulopathy and end-organ dysfunction, with mild inflammation, and was associated with higher 28-day mortality than class 1.^134^ Confirmation of this phenotype may suggest an important role for vascular dysfunction in the course and development of CARDS.

A longitudinal study of CARDS revealed no evidence of respiratory subphenotypes using cross-sectional data based on respiratory variables. Nevertheless, two subphenotypes developed during the first days of mechanical ventilation according to time-dependent analysis, with subphenotype 2 characterized by increasing minute ventilation, mechanical power, and ventilatory ratios.^135^ This finding reveals the importance of time as a critical variable in future subphenotype analyses of CARDS. Gattinoni L et al. classified two phenotypes of COVID-19 pneumonia, among which Type H is characterized by high elastance, high right-to-left shunting, high lung weight, and high recruitability, with 20–30% of patients meeting the severe ARDS criteria.^136^ These findings indicate that respiratory system elastance and recruitability could be signs of COVID-19-related phenotypes. Compared to ARDS patients, CARDS patients exhibited a greater median best PEEP, more collapse at low PEEP, and less hyperdistension at high PEEP.^137^

Many different phenotypes are likely to exist, overlapping in many respects and distinct in others. Based on the evidence of overlapping characteristics present in multiple subphenotypes, multimodal “omic” technologies would be beneficial for the accurate identification of ARDS subphenotypes. However, current evidence suggests that faster and more accurate identification of different phenotypes of ARDS is important for improving survival rates and prognosis, which also highlights the need for multimodal “omic” technologies.

### Biomarker-driven subphenotypes

Biological subtyping of patients with other pulmonary and nonpulmonary diseases could improve patient selection for clinical trials of targeted therapies.^138,139^ Wood SL et al. first grouped ARDS patients based solely on biomarker concentrations. Cluster analysis of 20 biomarkers of inflammation, coagulation, and endothelial activation was performed, and no clinical data or outcomes were evaluated. The results suggested that ARDS patients could be classified into two subphenotypes: the “uninflamed” phenotype and the “reactive” phenotype. Patients with a reactive phenotype had high plasma levels of markers of inflammation, coagulation, and endothelial activation (IL-6, IFN-γ, angiopoietin 1/2, and plasminogen activator inhibitor-1), with increased ICU mortality.^140^ The pathogenesis of ARDS differs between patients with pulmonary(ARDSp) and extrapulmonary(ARDSexp) lung injury, which is related to the identification of biomarker-driven subphenotypes. In ARDSp, the alveolar epithelium is primarily affected, while ARDSexp involves injury to the microvascular endothelial cells.^141^ Biomarkers can play a significant role in this differentiation, as they reflect the underlying pathophysiology. Calfee CS et al. demonstrated that biomarkers of lung epithelial injury (surfactant protein D (SP-D) and advanced glycation end product receptor (RAGE)) are significantly more abundant in ARDSp patients than in ARDSexp patients, while the level of an endothelial injury biomarker (Ang II) is considerably lower.^142^ Similarly, the concentrations of IL-1β and IL-18 in BALF were significantly greater in ARDSp patients than in ARDSexp patients.^143^ Variable ventilation strategies have demonstrated differential effects on biomarker expression in ARDSp and ARDSexp, suggesting that the mechanisms underlying respiratory improvement may vary between these subphenotypes.^144^ Sigh ventilation has been shown to reduce alveolar collapse and inflammation in pulmonary ALI, but its effects are more complex in extrapulmonary ALI, where it may increase markers of inflammation, apoptosis, and fibrogenesis.^145^

Gene expression also differs among ARDS subphenotypes. If differences in leukocyte gene expression are considered, the “reactive” subphenotype in ARDS patients with sepsis is characterized by neutrophil activation and oxidative phosphorylation, whereas the “uninflamed” subphenotype is characterized by enrichment of mitogen-activated protein kinase (MAPK) pathway.^146^ These results may explain the protective effect of simvastatin on the “hyperinflammatory” subphenotype,^147^ which is due to the antioxidative effect of simvastatin on endothelial cells^148^ and leukocytes.^149^

## Supportive therapy of ARDS

A recent report from an expert panel in the UK suggested that supportive management of patients with ARDS caused by COVID-19 should follow existing evidence-based ARDS guidelines.^150^ Here, we consider the evidence regarding the use of ventilation strategies, prone positioning, extracorporeal support, neuromuscular blockade (NMB), and corticosteroids for the management of ARDS. We also have summarized differences and up-to-date recommendations on supportive therapy for ARDS of recent American Thoracic Society (ATS)^151^ and European Society of Intensive Care Medicine (ESICM)^152^ clinical practice guidelines, shown in Table 2.

**Table 2:** ATS/ESICM clinical practice guidelines for supportive therapy in ARDS/CARDS

Management	Patients	Quality of Evidence (GRADE)	Strength of Recommendation	Comments	Future Research Priorities & Validations	Guideline
HFNO	AHRF	Moderate	Strong	Recommendation to reduce the intubation but no recommendation to reduce mortality.	Long-term functional outcome data; duration of HFNO.	ESICM
	AHRF from COVID-19	Low-moderate	Strong	Low level of evidence in favor for intubation and no recommendation; moderate level of evidence of no effect for mortality, for indirectness.		
CPAP/NIV	AHRF	Moderate-high	No recommendation	High level of evidence for mortality, moderate level of evidence for intubation.	Optimal indications for CPAP/NIV	ESCIM
	AHRF from COVID-19	Moderate	No recommendation	Weak recommendation to reduce intubation, but no recommendation to reduce mortality.		
Low tidal volume ventilation	ARDS	High	Strong	Use of low tidal volume ventilation strategies (i.e., 4–8 ml/kg PBW), compared to larger tidal volumes (traditionally used to normalize blood gases)	Merits of additional lung-protective strategies and personalized ventilator targets. investigation of VIVL.	ESCIM
	CARDS	Moderate	Strong			
Higher PEEP/FiO2 strategy	ARDS	High	No recommendation	No recommendation for or against routine PEEP titration with a higher PEEP/FiO2 strategy versus a lower PEEP/FiO2 strategy.	/	ESCIM
	CARDS	Moderate	No recommendation			
	ARDS	Moderate	Strong	Recommendation for using higher PEEP without LRMs rather than lower PEEP in patients with moderate to severe ARDS	Optimal strategy for setting PEEP; effect of PEEP strategies in specific populations and specific ARDS phenotypes.	ATS
PEEP titration guided principally by respiratory mechanics	ARDS	High	No recommendation	No recommendation for or against PEEP titration guided principally by respiratory mechanics, compared to PEEP titration based principally on PEEP/FiO2 strategy.	Individual effect of different levels of PEEP; hemodynamic cost of higher PEEP; Esophageal pressure-guided PEEP and distending pressure.	ESCIM
	CARDS	Moderate	No recommendation			
Prolonged high-pressure recruitment maneuvers	ARDS	Moderate	Strong	Recommendation for against use of prolonged high-pressure recruitment maneuvers (defined as airway pressure maintained ≥ 35 cmH2O for at least one minute).	/	ESCIM
	CARDS	Low	Strong			
	ARDS	Moderate	Strong	Recommendation for against using prolonged (PEEP ⩾35 cm H2O for >60 s) LRMs in patients with moderate to severe ARDS	/	ATS
Brief high-pressure recruitment maneuvers	ARDS	High	Weak	Recommendation for against routine use of brief high-pressure recruitment maneuvers (defined as airway pressure maintained ≥ 35 cmH2O for less than one minute).	Safety risks; frequency and benefit group.	ESCIM
	CARDS	Moderate	Weak			
Prone position	ARDS	High	Strong	Recommendation for using prone position as compared to supine position for patients with moderate-severe ARDS (defined as PaO_2_/FiO_2_ < 150 mmHg and PEEP ≥ 5 cmH2O, despite optimization of ventilation settings)	Trials in moderate-severe CARDS	ESCIM
	CARDS	Moderate	Strong			
Time of prone position	ARDS	High	Strong	Recommendation for starting prone position in patients with ARDS receiving invasive mechanical ventilation early after intubation, after a period of stabilization during which low tidal volume is applied and PEEP adjusted and at the end of which the PaO2/FiO2 remains < 150 mmHg.	Different durations of prone position; guidance on when to cease prone position.	ESCIM
	CARDS	Moderate	Strong			
Awake prone positioning	AHRF	No evidence	No recommendation	Recommendation for awake prone positioning as compared to supine positioning for non-intubated patients with AHRF./	The location (ICU vs non-ICU), the optimal respiratory support (HFNO, CPAP, NIV), and the impact of APP on inspiratory effort, work of breathing, and potential lung injury.	ESCIM
	AHRF from COVID-19	Low-moderate	No recommendation			
NMBA	ARDS	Moderate	Strong	Recommendation for against the routine use of continuous infusions of NMBA in patients with moderate-to-severe ARDS.	Successful extubation, re-intubation, paralysis recall, ICU acquired weakness and health-related quality of life and the specific role of NMBA in a prone position; patient-ventilator interaction; views of patients and caregivers.	ESCIM
	CARDS	No evidence	No recommendation			
	Early ARDS (≤48 h of MV) with PaO_2_/FiO_2_ ≤ 100	Low	Conditional	Uncertainty around the harms of the concomitant sedation required with NMBA.	Patient-ventilator interaction; NMBA agent selection; the impact of the timing of initiation, dosing, and duration; Longitudinal data of NMBAs on long-term outcomes.	ATS
VV-ECMO	ARDS	Moderate	Strong	Patients with severe ARDS as defined by the EOLIA trial eligibility criteria; ECMO center should meet defined organizational standards; management strategy similar to that used in the EOLIA trial.	Long-term multidimensional outcomes for patients and families; ECMO-specific morbidities	ESCIM
	CARDS	Low	Strong			
	ARDS (PaO_2_/FiO_2_ ≤ 80 or PH <7.25 with pCO_2_ ≥ 60)	Low	Conditional	Limitations of available data and practical concerns; less invasive therapies before the consideration of VV-ECMO; focus on individuals most likely to benefit; high-volume, dedicated ECMO center.	Long-term outcomes in ECMO survivors; appropriate supportive measures for patients receiving ECMO；the impact of ECMO on resource allocation	ATS
ECCO_2_R	ARDS	High	Strong	Recommendation against the use of ECCO_2_R for the treatment of ARDS to prevent mortality outside of randomized controlled trials	Response to ECCO_2_R in a specific population of ARDS patients	ESCIM
	CARDS	Moderate	Strong			
Corticosteroids	ARDS (PaO_2_/FiO_2_ ≤ 300)	Moderate	Conditional	The initiation of corticosteroid treatment >2 weeks after the onset of ARDS may be associated with harm; close surveillance for adverse effects is needed in particular patients.	Optimal corticosteroid regimen; effects on different subpopulations of ARDS patients.	ATS

Current ATS/ESCIM guidelines for the management of acute respiratory distress syndrome, including ventilation strategy, *ECMO* neuromuscular blocking agents, and Corticosteroids

*ATS/ESICM American Thoracic Society and European Society of Intensive Care Medicine, *ARDS* acute respiratory distress syndrome, *CARDS* COVID-19 related acute respiratory distress syndrome, *HFNO* high-flow nasal cannula oxygen, *AHRF* acute hypoxemic respiratory failure, *CPAP/NIV* continuous positive airway pressure and non-invasive ventilation, *PBW* predicted body weight, *VIVL* ventilator-induced lung injury, *PEEP* positive end-expiratory pressure, *FiO*_*2*_ fraction of inspired oxygen, *PaO*_*2*_ partial pressure of oxygen, *LRM* lung recruitment maneuvers, *ICU* intensive care unit, *APP* awake prone positioning, *NMBA* neuromuscular blocking agent, *MV* mechanical ventilation, *VV-ECMO* venovenous extracorporeal membrane oxygenation, *EOLIA* *ECCO*_*2*_*R* extracorporeal carbon dioxide removal

### Ventilation strategies

Noninvasive respiratory support, rather than conventional oxygen therapy, might be considered as the initial respiratory management approach for adult patients with acute respiratory failure who are suspected of having ARDS if there are no contraindications for noninvasive respiratory support and if organ failure other than respiratory failure is absent.^153^ Patients on a high-flow nasal cannula oxygen (HFNO) or noninvasive ventilation (NIV) should be carefully managed in an environment where tracheal intubation can be conducted after the start of noninvasive respiratory support.

#### High-flow nasal cannula oxygen

High-flow nasal cannula oxygen has gained traction over the past decade, in large part due to a multicentre trial showing decreased mortality compared with noninvasive positive pressure ventilation (NPPV) and standard oxygen therapy in patients with acute hypoxemic respiratory failure,^154^ as well as improved patient comfort compared with NIV and invasive mechanical ventilation (IMV). Clinical practice guidelines have strongly endorsed HFNO therapy over standard oxygen therapy for patients with hypoxemic respiratory failure, as its use has been associated with reduced intubation rates and reduced escalation of respiratory support.^155^ HFNO therapy may be considered for patients with ARDS if the airway protective reflex is intact and the patient has stable hemodynamics. The benefits of this approach include decreased respiratory effort due to the washout of the anatomic dead space.

#### Noninvasive ventilation

Noninvasive ventilation is frequently used in patients with ARDS, although its use remains controversial. The potential benefits include the avoidance of ventilator-associated events and the need for deep sedation, which often occurs with IMV. In addition, appropriately titrated end-expiratory pressure could decrease injury related to vigorous spontaneous breathing.^156^ Potential harms include delayed (necessary) intubation, inability to control tidal volumes and monitor airway pressures, and an inconsistent mask seal, which could lead to cyclic recruitment-derecruitment of lung units, causing atelectrauma. A small randomized trial using a full-helmet interface in patients with moderate to severe ARDS showed reductions in the rates of intubation and mortality compared with those of standard facemask NIV.^157^ A study using a helmet interface (compared with HFNO) in patients with CARDS did not show a mortality benefit. Nevertheless, it did demonstrate reductions in intubation rate and ventilator days.^158^ Helmet NIV, which requires familiarity with the technology, is not routinely available in most hospitals and would benefit from further study in real-world settings before its widespread use.

#### Management with invasive mechanical ventilation

##### Lung-protective ventilation

Lung-protective ventilation (i.e., tidal volumes of <6 ml/kg of predicted body weight and plateau pressure ≤30 mm Hg) is a key recommendation based on the findings of the landmark ARMA trial,^159^ which showed reduced mortality and increased ventilation-free days. RCTs of novel ventilatory strategies have continued to reinforce the benefit of lung-protective ventilation.^160–162^

##### PEEP

PEEP is the pressure that maintains some degree of inflation during the end-expiratory pause. Higher PEEP increases the mean airway pressure, which usually improves oxygenation. And maintaining inflation during exhalation also decreases atelectrauma.^163^ The most commonly used method for PEEP selection is to apply an algorithm matching PEEP to the FiO2 the patient requires,^159^ which was tested in clinical trials by the ARDS network (ARDS Net) in the USA and is relatively simple to apply: the higher the fraction of oxygen required is, the higher the selected PEEP is. Three large trials^122,164,165^ tested the hypothesis that a higher-PEEP protocol would improve survival compared with the traditional ARDS Net PEEP protocol. However, no substantial differences in clinical outcomes were observed in any of the three trials, suggesting that a high-PEEP strategy was not superior for all patients with ARDS. Another trial applying an aggressive high-PEEP strategy plus high-pressure recruitment maneuvers revealed a statistically significant increase in mortality in the intervention arm.^166^ Therefore, heterogeneity of individual patient responses to PEEP strategies is recognized,^167^ leading to increasing interest in personalized PEEP strategies, although these strategies have not been demonstrated to yield additional benefits over conventional PEEP strategies.^166,168^

##### Driving pressure

Driving pressure (i.e., plateau pressure minus end-expiratory pressure) might be an independent predictor of survival in patients with ARDS.^160^ Amato et al. demonstrated that driving pressure was the key mediator of the benefits of PEEP and tidal volume strategies. An upper limit of 15 cm H2O for driving pressure is recommended, which could cause considerable lung stress^169^ and increased mortality.^23,160^ Conversely, two clinical trials revealed increased mortality in the setting of lower driving pressure (i.e., ≤15 cm H2O), suggesting that driving pressure might not be as valuable as initially expected for predicting mortality.^166,170^

##### Mechanical power

Mechanical power is a novel concept applied in the context of ARDS. MP refers to the amount of energy per unit of time transmitted to the respiratory system by a mechanical ventilator, as determined by volume, pressure, flow, and respiratory rate.^171,172^ MP might be a better driver of lung-protective ventilation than individual ventilator parameters, as it considers the balance of several parameters as a whole.^173^ Reanalyses of clinical trials and observational data showed that MP was associated with increased mortality.^174,175^ In a retrospective analysis of 8207 patients, a consistent increase in the risk of death was observed with an MP greater than 17 J/min.^176^ The complexity of interpreting MP limits its clinical use. However, recently, it was found that the driving pressure and RR components of MP were the best predictors of mortality.^161^ Although these variables can be easily measured at the bedside, the additional benefits of MP remain uncertain.

#### Prone positioning

In the supine position, the V˙/Q˙ mismatch leads to poor oxygenation. Shifting to the prone posture has several advantages, including reversal of the gravitational forces that move surrounding structures such as the heart and diaphragm, ultimately leading to more homogeneous lung perfusion. Starting from the observation that oxygenation improved in patients in the prone position, studies identified several physiological mechanisms underlying this improvement, including a decrease in the differential distribution of ventilation between the ventral and caudal lung regions and a shift in the density distribution of the edematous lung, increasing the V˙/Q˙ ratio.^177,178^ A series of randomized trials^179–182^ paralleled the evolution of this pathophysiological understanding. However, none of these trials individually showed a survival benefit of prone positioning; post hoc analysis suggested a potential benefit for the most severely hypoxemic patients when the prone position was combined with low stretch ventilation and applied for more extended periods (16 h).^183^ Based on these findings, a prospective study examined prone ventilation for 17 hours daily for patients with moderate or severe ARDS and showed a statistically significant survival benefit.^184^ Thus, the prone position should be strongly considered for patients meeting certain criteria (PaO2/FiO2 ratio persistently <150) without contraindications.

During the COVID-19 pandemic, prone positioning was used successfully in awake, non-intubated patients with acute hypoxemic respiratory failure.^185,186^ However, its benefit remains uncertain, with conflicting findings from clinical trials.^187,188^ As an adjunct to extracorporeal membrane oxygenation (ECMO) therapy, which is safe and effective, the use of the prone position was associated with a greater probability of surviving and being weaned off of ECMO at 90 days in a clinical trial.^189^ In another RCT involving 170 patients, prone positioning did not significantly reduce the time to successful weaning of ECMO compared with supine positioning in patients with severe ARDS supported by VV-ECMO.^190^

Complications resulting from prone positioning are rare. The PROSEVA study revealed no significant differences between groups in terms of nonscheduled extubation, hemoptysis, mainstem bronchus intubation, or cardiac arrest.^184^ A meta-analysis of eight RCTs revealed that patients with ARDS who underwent prone positioning had greater rates of endotracheal tube obstruction and pressure sores.^191^ Awake-prone positioning is also safe, with studies demonstrating no increased risks of complications or slightly increased rates of skin breakdown, line dislodgement, back pain, or generalized discomfort.^187,192,193^ However, careful attention must be given to the proning procedure to avoid disruption of vascular access catheters and endotracheal tubes and, while the patient is prone, to avoid pressure-related complications.

### Neuromuscular blockade

The use of NMB in patients with moderate to severe ARDS has the potential benefit of decreasing ventilator-associated lung injury and improving mortality at 28 days without increasing the incidence of neuromuscular weakness.^194^ A large randomized study identified an adjusted mortality advantage of NMB (cisatracurium) in deeply sedated patients with moderate or severe ARDS.^195^ In addition, subsequent trials failed to show survival benefits in patients with moderate or severe ARDS who were randomly assigned to receive cisatracurium with deep sedation for 48 h compared with light sedation if tolerated and goal-oriented sedation if not tolerated.^196,197^ Importantly, in both trials, the duration of NMB in the study protocol was intentionally short (≤48 h), with no difference in the incidence of ICU-acquired weakness with NMB. Although NMB is thus not mandated for patients with moderate or severe ARDS, bolus and/or short-duration infusions of NMB agents are safe and could improve gas exchange and ventilator synchrony.

NMB must be used cautiously for patients who are unable to achieve ventilation synchrony within lung-protective targets, for patients with severe hypoxemia despite deep sedation, and for patients whose plateau pressures are high or difficult to measure accurately. Once initiated, clinicians should consider daily whether NMB remains helpful and consider discontinuation at the earliest opportunity.

### Extracorporeal support

During the era of lung-protective ventilation, two RCTs, CESAR^198^ and EOLIA,^199^ investigated the role of venovenous ECMO for severe ARDS patients, with somewhat conflicting results. A post hoc Bayesian analysis showed a high probability that early ECMO was beneficial.^200^ Furthermore, in a subsequent meta-analysis of individual patient data, including both the CESAR and EOLIA RCTs, the precision of the treatment effect improved (combined data for 429 patients). A statistically significant improvement in 90-day mortality was observed in the ECMO group.^201,202^ Extracorporeal carbon dioxide removal (ECCO2R) is a low-flow form of venovenous support that has been studied in ARDS patients. The REST trial,^203^ which investigated ECCO2R in patients with acute hypoxaemic respiratory failure, revealed no difference in 90-day mortality, and there was an increased incidence of serious adverse events, including clinically significant bleeding, the need for more sedation and NMB and a longer duration of mechanical ventilation, in the ECCO2R group. On this basis, the use of ECCO2R for the treatment of ARDS is not recommended outside RCTs.

Evidence of the benefit of ECMO has been extended to COVID-19 patients.^204^ A comparative effectiveness study including 844 of 7345 eligible patients (11.5%) with COVID-19-associated respiratory failure who received ECMO in five countries was recently conducted; the results indicated that ECMO was associated with reduced mortality compared with that of supportive therapy.^205^ Although confirmation in an RCT would be desirable, these findings provide reassurance regarding the use of ECMO in a select population of patients with severe COVID-19.

Taken together, these data suggest that patients with severe ARDS could benefit from treatment with ECMO. Notably, patients receiving ECMO should receive an overall management strategy similar to that used in the EOLIA trial or comply with the criteria for ECMO defined by expert groups.^206^

### Corticosteroids

Steroids have potent anti-inflammatory effects that could benefit patients with ARDS, but the role of corticosteroids in ARDS management has long been controversial. An early trial investigating the use of methylprednisolone (MPS) in patients with persistent ARDS revealed an association with an increased risk of late mortality (i.e., day 60 and day 180) when steroids were initiated beyond day 14 after ARDS onset.^207^ A multicentre trial investigated a high dose (i.e., 20 mg once daily for five days) followed by a lower dose (i.e., 10 mg once daily for five days) of dexamethasone, indicating that early administration of dexamethasone could reduce the duration of mechanical ventilation and overall mortality in patients with established moderate-to-severe ARDS.^208^ Consistently, a subsequent meta-analysis of 999 patients from eight RCTs with ARDS revealed a mortality benefit of corticosteroid use.^209^ However, steroid regimens differed between studies (e.g., different types, doses, and durations), and there were differences in the patient populations investigated (e.g., early vs. late ARDS and some studies were performed before lung-protective ventilation).

Corticosteroid use has increased after positive results of clinical trials of dexamethasone in patients with COVID-19 pneumonia,^210^ and a subsequent meta-analysis reported similar results.^211^ There was evidence of an association of corticosteroid use with hyperglycemia, but no specific evidence supports concerns about other adverse events. However, we must note that the results of the study performed by Moreno G showed that the use of glucocorticoids as coadjuvants was significantly associated with increased ICU mortality in patients with severe influenza pneumonia, suggesting that corticosteroids should not be used as coadjuvant treatment for patients with influenza pneumonia.^212^ Therefore, the potential benefits and harms of corticosteroid use in ARDS patients, especially those with influenza pneumonia-induced ARDS, still need stronger clinical evidence.

## Cell therapy

In recent years, cell therapy has shown great promise in preclinical ARDS research. A wide range of cells have been identified as potential candidates, especially mesenchymal stromal cells whose therapeutic potential in treating ARDS has been confirmed in multiple preclinical studies and even clinical trials. The therapeutic effects of these cells mainly occur via two different mechanisms: direct cell interactions and the paracrine release of cellular components such as extracellular vesicles (EVs). Here, we summarize the latest mechanisms of different cell therapies for ARDS.

### MSC-based cell therapy

#### MSC-based cell therapy in preclinical studies

**Fig. 4:**
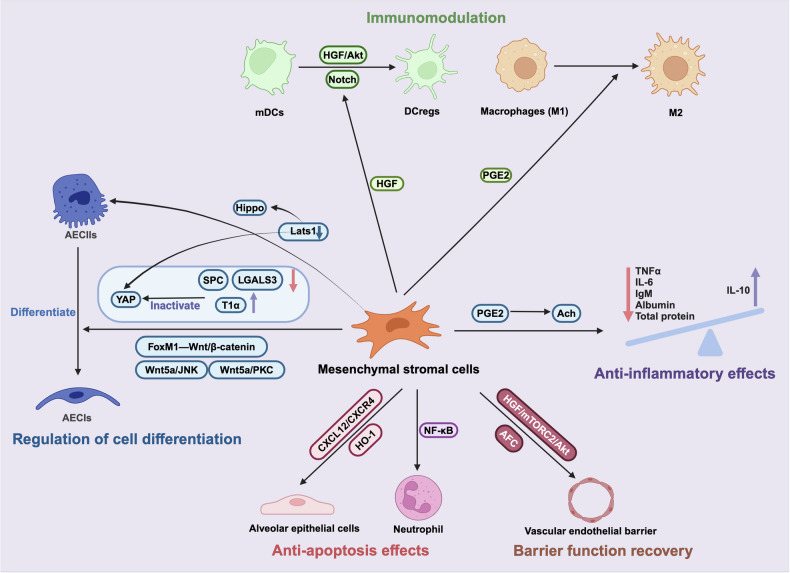
**MSC-based cell therapy in ARDS**. MSCs have demonstrated strong capabilities in the treatment of ARDS, mainly including regulating the differentiation of alveolar epithelial cells, activating immune cells such as dendritic cells and macrophages to modulate immune function, repairing barrier function, and exerting anti-inflammatory and anti-apoptotic effects. *AECII type II alveolar epithelial cells, AECI type I alveolar epithelial cells, YAP Yes-associated protein, SPC surfactant protein C, LGALS3 galectin3, T1α podoplanin, FoxM Forkhead box (Fox) transcription factor family, DCs dendritic cells, HGF hepatocyte growth factor, PGE prostaglandin, Ach acetylcholine, AFC alveolar fluid clearance, CXCL C-X-C motif ligand, HO-1 heme oxygenase-1

Several animal studies have demonstrated that MSC therapy is a promising novel intervention for ARDS. The actions of MSCs involve several mechanisms, which are described in Fig. 4.

##### Anti-inflammatory effects

The anti-inflammatory pathway is a key mechanism underlying the treatment of ARDS with MSCs. After autologous transplantation of bone marrow-derived mesenchymal stromal cells (BM-MSCs) into sheep, decreases in total inflammatory cell numbers, neutrophil numbers, macrophage numbers, proinflammatory cytokine levels (IL-6 and tumor necrosis factor-alpha (TNF-α)), and total protein, IgM, and albumin levels were observed, while the level of an anti-inflammatory cytokine (IL-10) increased. BM-MSC transport can reduce local and systemic levels of inflammatory factors.^213,214^ Both adipose-derived mesenchymal stromal cells (AD-MSCs) and umbilical cord-derived mesenchymal stromal cells (UC-MSCs) attenuate inflammation and modulate inflammatory factors.^215,216^ Recently, Zhang X et al. proposed a novel potential anti-inflammatory mechanism of MSCs in ARDS involving the cholinergic anti-inflammatory pathway (CAP). The therapeutic efficacy of BM-MSCs can be significantly reduced by blocking the vagus nerve, administering drug inhibitors, or implementing gene knockout to inhibit CAP, and BM-MSC-derived prostaglandin E2 (PGE2) can promote acetylcholine (Ach) synthesis and release. Based on the efficacy of nAChR and α7nAChR agonists, a clinical study revealed that lobeline, a nicotinic cholinergic receptor agonist, attenuated pulmonary inflammation and alleviated respiratory symptoms in ARDS patients.^24^

##### Regulation of cell differentiation

In the late phase of ARDS, type II alveolar epithelial cells (AECIIs) differentiate to repair damaged type I alveolar epithelial cells (AECIs) due to their high progenitor cell capacity.^217,218^ FoxM1 is a member of the Forkhead box (Fox) transcription factor family, which is crucial for the differentiation of AECIIs into AECIs after ALI.^219,220^ BM-MSCs overexpressing FoxM1 attenuate pulmonary edema and fibrosis, mitigate oxidative damage and inflammatory responses, and restore vascular integrity^221^ via the Wnt/β-catenin pathway.^222^ Yes-associated protein (YAP) is also an essential molecule for the differentiation of AECIIs into AECIs. In particular, hUC-MSCs could inactivate YAP on AECIIs, thus inducing AECII differentiation into AECIs.^223^

In addition to promoting the differentiation of AECIIs into AECIs, MSCs can also differentiate directly into AECIIs, which is associated with the Hippo signaling pathway. Lats1 knockdown inhibits Hippo signaling activity in mBM-MSCs, thus increasing the retention of mBM-MSCs in ARDS lung tissue. mBM-MSCs with downregulated Hippo signaling differentiated into alveolar epithelial cells, reinforcing alveolar epithelial tight junctions.^224^ Significantly, when Lats1 activity decreases, YAP remains unphosphorylated, which stimulates the proliferative and antiapoptotic effects of the Hippo signaling pathway, thus promoting cell proliferation.^225^ In addition, activation of the noncanonical Wnt5a/JNK and/or Wnt5a/PKC pathway(s) promotes the differentiation of BM-MSCs into AECIIs and promotes their migration to injured lung tissue.^226^ The migration ability and homing ability of BM-MSCs in damaged lung tissue could also be enhanced by Vimentin-Rab7a and ROR2.^227,228^

##### Immunomodulation

HGF-overexpressing MSCs promote the differentiation of mature DCs (mDCs) into tolerogenic dendritic cells (DCregs) via direct cell‒cell contact, thus reducing ALI through the HGF/Akt pathway and the Notch pathway.^229,230^ Additionally, PGE2-primed hP-MSCs promote the polarization of macrophages from the M1 to M2 and regulate cytokine production, exerting strong protective effects against LPS-induced ALI.^231^ Preconditioning MSCs before administration alleviates ARDS by enhancing their immunomodulatory capacity, for example, BM-MSCs preconditioned with ARDS serum exert stronger immunotherapeutic effects through VEGF and PGE2.^232^

##### Anti-apoptosis effects

Alveolar epithelial injury is the primary pathophysiological mechanism underlying ARDS. BM-MSCs inhibit the apoptosis of alveolar epithelial cells in mice with ALI through the CXCL12/CXCR4 signaling axis.^233^ MSC-CM alleviates neutrophil apoptosis in the context of endotoxin-induced ALI by inhibiting the NF-κB pathway.^234^ BM-MSCs overexpressing HO-1 could improve the survival rate and attenuate lung impairment in ALI rats by inhibiting apoptosis and enhancing the paracrine effects of MSCs.^235^

##### Administration

One study compared different routes of stem cell administration; endobronchial and intravenous administration were equally effective for treating ARDS in sheep. The authors observed by endobronchial [^18^F]-FDG that labeled cells were trapped inside the lung with no systemic distribution. Administration via the intravenous route leads to a systemic distribution of the cells, which mainly lodge in the lung.^236^ Interestingly, whether MSC therapy is beneficial depends on the lung microenvironment at the time of administration. The results of one study showed that MSCs may have detrimental effects on the lung environment, inducing high levels of both IL-6 and fibronectin (FN) and low antioxidant capacity. Improving the lung environment before MSC administration could enhance the lung-protective effects of MSCs.^237^

##### Gene regulation

A study profiled the microRNAome and transcriptome of lungs from mice with endotoxin-induced ALI that were treated with either a placebo or MSCs and showed that MSCs regulate the expression of miR-27a and its various target genes, including VAV3, which is involved in sepsis-induced lung injury. In addition, increased miR-27a-5p expression was observed in patients who died from ARDS and had autopsy-proven diffuse alveolar damage (DAD) compared with patients who did not have DAD.^238^ BM-MSC-CM decreased miR-193b-5p expression and increased occludin protein expression in the lungs of septic mice model, and alleviated the loss of barrier function. Importantly, miR-193b-5p expression was increased and occludin protein expression was markedly decreased in lung autopsy samples from ARDS patients compared to those from non-ARDS controls. In addition, a study compared the minicircle DNA system to a conventional plasmid vector for the transfection of BM-MSCs; the results showed that minicircles can achieve more efficient and sustained expression of ANGPT1, further enhancing the therapeutic effect in an ALI mice model.^239^

##### Barrier function recovery

Hepatocyte growth factor (HGF) is required for the ability of BM-MSCs to restore lung permeability and ameliorate lung injury induced by LPS.^240^ HGF secreted by BM-MSCs promotes vascular endothelial barrier recovery via activation of the mTORC2/Akt pathway.^241^ TEER measurements, permeability assays, and immunohistological findings revealed that AD-MSCs enhance the barrier function of lung epithelial cells in vitro.^242^ Interestingly, BM-MSCs preconditioned by mechanical stretching in vitro also restore the permeability of endothelial cells treated with LPS.^243^ Moreover, BM-MSCs had the ability to restore AFC in an ex vivo perfused human lung, which was partially attributed to the increased sodium-dependent resolution of alveolar edema.^244,245^

#### MSC -based cell therapy in clinical studies

##### Clinical therapy with MSCs in ARDS patients

To date, many clinical studies have demonstrated the benefits of different MSC treatments in ARDS patients, as indicated by reduced inflammation and alveolar permeability and improved lung function, including tidal volume and compliance.^246–248^ Most trials have used 10^6^ cells/kg, with the highest dose of 10^7^ cells/kg being safe. Although some adverse events have been observed in trials, it has been proven that there is no correlation with MSC treatment. Notably, Simonson OE et al. described a 5-year follow-up of 2 patients with severe refractory ARDS who received a single intravenous injection of BM-MSCs at a dose of 2 × 10^6^ cells/kg, including health-related quality of life (HRQoL), physical capacity, and pulmonary morphology and function. Both patients fully achieved a full physical and mental recovery.^248,249^ However, extensive studies with long follow-up periods are needed to confirm the safety of these treatments because of the small sample size. We look forward to the results of an ongoing phase III clinical trial of MSC therapy for ARDS. Additionally, the viability and efficacy of MSCs require more attention. More details are shown in Table 3.^215,246–253^

**Table 3:** Clinical therapy of MSCs on ARDS

MSCs resource	Clinical phase	Study group	Case	Dose	Delivery method	Delivery time	Outcome measures	Effect	Trial registration	Ref
BM-MSCs	A case series	Severe ARDS	2	2 × 10^6^ cells/kg at a single infusion	Intravenously	Deteriorated with ECMO support	Clinical Outcomes: BAL fluid and serum biomarkers. CXRs and CT scans.	Improved lung function, with overall progressive clinical improvement; recovered physical and mental capacities.	/	^248,249^
UC-MSCs	A case series	Severe ARDS	1	1.0 × 10^6^ cells/kg at a single infusion	Intratracheally	On hospital day 114	/	An improvement in mental status, lung compliance, PaO2/FiO2 ratio, and CXRs.	/	^250^
BM-MSCs	Phase I	Moderate- severe ARDS	9	10^6^/kg or 5 × 10^6^/kg or 10 × 10^6^/kg cells	Intravenously	Within 120 hours of meeting the Berlin definition for ARDS	Primary outcomes: pre-specified infusion-associated events and serious adverse events; Secondary outcomes: clinical improvements and laboratory testing and mechanistic analyses.	Favorable tolerability and short-term safety profile of MSCs.	NCT01775774	^215^
UC-MSCs	Phase I	Moderate-severe ARDS	9	1, 5, or 10 × 10^7^ cells/kg at a single infusion	Intravenously	Over 1 hour within 48 hours of enrollment	Primary outcomes: the safety and tolerability of transfusion; Secondary outcomes: all serious adverse events and nonserious adverse events.	Circulating inflammatory biomarkers and MSC markers were notably reduced, whereas the immune cell markers were increased after cell infusion.	ISRCTN52319075	^246^
UC-MSCs	Phase I	Moderate-severe ARDS	9	100, 200, or 400 × 10^6^ cells at a single infusion	Intravenously	Within 48 h from the onset of ARDS	Primary outcomes: serious adverse events; Secondary outcomes: clinical improvements	400 × 10^6^ cells of ORBCEL-C was safe and feasible in critically ill patients with moderate to severe ARDS.	NCT03042143	^251^
AD-MSCs	Phase I	Mild, moderate, and severe ARDS	6	1 × 10^6^ cells/kg at a single infusion	Intravenously	Diagnosed within 48 hours	Primary outcomes: adverse events; Secondary outcomes: clinical improvements and laboratory testing and mechanistic analyses.	Decreased epithelial cell injury as evidenced by the decrease of SP-D level.	NCT01902082	^252^
BM-MSCs	Phase IIa	Moderate-severe ARDS	17	10 × 10^6^/kg cells at a single infusion	Intravenously	After clinically stable for 2 h	Primary outcomes: the safety of the MSC infusion, assessed with prespecified infusion-associated adverse events; Secondary outcomes: clinical improvements.	Clinical outcomes did not differ significantly between groups, but MSCs reduced lung injury biomarkers, including Ang-2, IL-6, and sTNFR-1, with a trend for improvement in the oxygenation index.	NCT02097641	^247,253^

The efficacy of MSC in treating ARDS has been explored in clinical applications. All trials showed significant safety, and most benefited from improvements in lung function and reductions in inflammatory factors

*MSCs mesenchymal stromal cells, *ARDS* acute respiratory distress syndrome, *BM-MSCs* bone marrow-derived MSCs, *Ang-2* Angiopoietin-2, IL-6 interleukin-6, *sTNFR-1* soluble TNF receptor-1, *BAL* bronchoalveolar lavage, *CXRs* chest radiographs, *UC-MSCs* umbilical cord-derived MSCs, *PaO*_*2*_*/FiO*_*2*_ arterial pressure of oxygen/inspiratory fraction of oxygen, *ORBCEL-C* a population of CD362 enriched umbilical cord-derived MSCs, *AD-MSCs* adipose-derived MSCs, *SP-D* surfactant protein D

##### Clinical therapy with MSCs in CARDS patients

Although MSCs have been used to treat various diseases without safety concerns, these results may not be directly applicable to patients with COVID-19. Several trials have confirmed the safety of MSCs for treating SARS-CoV-2-induced ARDS, and MSC therapy has also been shown to improve the survival rates and clinical outcomes of patients.^254–256^ However, no efficacy of MSCs in patients with CARDS was demonstrated in two trials.^257,258^ Notably, patients who develop sepsis or multiorgan failure may not be good candidates for stem cell therapy because the death occurred 5-19 days after the first MSC infusion in four patients with multiorgan failure or sepsis.^259^ More details of these trials are shown in Table 4.^254–267^

**Table 4:** Clinical therapy of MSCs on CARDS

MSCs resource	Clinical phase	Study group	Case	Dose	Delivery method	Delivery time	Outcome measures	Effect	Trial registration	Ref
UC-MSCs	Phase IIb	Mild-severe CARDS	21	10^6^ cells/kg at three infusions	Intravenously	Diagnosed for < 96 h	Primary outcomes: respiratory improvement assessed; secondary outcomes: clinical improvements.	D0-to-D7 PaO2/FiO2 changes did not differ significantly.	IRCT20200621047859N4	^257^
PL-MSCs	Phase I	Moderate-severe CARDS	10	10^6^ cells/kg at a single infusion	Intravenously	More than 96 h have passed since the diagnosis of CARDS	Main outcomes: the safety of transplantation.	The mean length of hospitalization, serum oxygen saturation, and other clinical and laboratory parameters were not significantly different.	NCT04333368	^258^
BM-MSCs	Phase I/II	Severe CARDS	8	3.15 ×10^6^cells/kg at three infusions	Intravenously	Within 2 days of ICU admission	Safety outcomes: tolerability and adverse events related to MSCs; efficacy outcomes.	Higher survival in the MSC cohort; no significant difference in the need for mechanical ventilation nor the number of invasive ventilation-free days, high flow nasal oxygenation-free days, oxygen support-free days and ICU-free days.	NCT04445454	^254^
UC‐MSCs	Phase I/IIa	Mild-severe CARDS	12	100 ± 20 × 10^6^cells at two infusions	Intravenously	At day 0 and day 3	Primary outcomes: safety, adverse events; Secondary outcomes: clinical improvements and laboratory testing and mechanistic analyses.	Inflammatory cytokines were significantly decreased, and survival and time to recovery were improved in the MSC cohort;	NCT04355728	^255,260^
BM-MSCs	A case series	Moderate-severe CARDS	11	2 × 10^6^ cells/kg at two infusions	Intravenously	Given 48–120 h apart	Primary outcomes: ICU mortality; Secondary outcomes: clinical improvements.	Improved liberation from mechanical ventilation and discharge from the ICU and/or hospital with significantly declined CRP levels；	/	^256^
UC-MSCs/ PL-MSCs	A case series	Mild-severe CARDS	11	200 × 10^6^cells at three infusions	Intravenously	Every other day	Main outcomes: the safety and potential adverse events;	Reduced dyspnea and increased SpO2 were observed with decreased inflammatory biomarkers including TNF-α, IL-8, IL-6, CRP, IFN-γ; Four patients with multi-organ failure or sepsis died in 5-19 days after the first MSC infusion.	/	^259^
UC-MSCs	A case series	Moderate-severe CARDS	13	10^6^ cells/kg at three infusions	Intravenously	On treatment days 0, 3, and 6	Safety and clinical improvements	Decreased inflammatory markers, more rapid recovery of blood lymphocytes, and reduced SP-D	/	^261^
UC-MSCs	A case series	Severe CARDS	5	10^6^ cells/kg at a single infusion	Intravenously	/	Adverse events and clinical improvements	Improved respiratory function, anti-inflammatory capacity	/	^262^
UC-MSCs	Phase I	Severe CARDS	20	10^6^ cells/kg at a single infusion	Intravenously	On day 8 (ranged from day 2‐30) of treatment in the ICU	Primary outcome: survival rate and/or length of ventilator usage; clinical and laboratory improvement, with serious adverse events	Increased the survival rate; anti-inflammatory by decreasing IL-6	NCT04457609	^263^
PL-MSCs	A case series	Moderate to severe CARDS	5	200 ± 20 × 10^5^cells at two infusions	Intravenously	on Days 0 and 4.	Safety and clinical efficacy.	Suppressed hyper-inflammatory states	/	^264^
BM-MSCs	Phase II/III	Moderate to severe CARDS	112	2 × 10^6^ cells/kg at two infusions	Intravenously	The second infusion 4 days after the first infusion	Primary outcomes: reduction in all-cause mortality within 30 days; secondary outcomes: days alive off mechanical ventilation within 60 days.	MSCs did not improve 30-day survival or 60-day VFD in patients with moderate and/or severe CARDS.	NCT04371393	^265^
UC-MSCs	Phase I	Non-severe CARD	10	1 × 10^6^ cells/kg at three infusions	Intravenously	Every other day (days 1, 3 and 5)	Primary outcome: the safety of three doses; secondary outcomes: changes in specific biomarkers of inflammatory dysregulation.	MSC improved respiratory function and cytokine storm reduction in CARDS and increased the number of injections can increase patients’ recovery.	IRCT20160809029275N1	^266^
Decidua-MSCs	A case series	Mild-severe CARDS	10	1 × 10^6^ cells/kg at 1-2 infusions	Intravenously	After 3 days in patients needing more than one dose	Safety and efficacy by survival, oxygenation and effects on levels of cytokines	Improved oxygenation, decreased inflammatory cytokine levels and cleared pulmonary infiltrates in CARDS.	/	^267^

The effect of MSC therapy on CARDS patients was investigated in clinical trials. 10^6^ cells/kg is the usual dose for MSCs infusion, with considerable safety. However, it may not suitable for all CARDS patients, especially those who develop sepsis or multi-organ failure

*MSCs mesenchymal stromal cells, *CARDS* COVID-19-associated ARDS, *UC-MSCs* umbilical cord-derived MSCs, *PaO*_*2*_*/FiO*_*2*_ arterial pressure of oxygen/inspiratory fraction of oxygen, *PL-MSCs* placenta-derived MSCs, *BM-MSCs* bone marrow-derived MSCs, *CPR* C-reactive protein, *SpO*_*2*_ oxygen saturation, *TNF* tumor necrosis factor, *IL* interleukin, *IFN* interferon, *SP-D* surfactant protein D, *VFD* ventilator-free days, *ICU* intensive care unit, *MV* mechanical ventilation, *HFNO* high flow nasal oxygen

### Non-stem cell-based cell therapy in ARDS patients

In addition to stem cell treatment, several cell types, such as alveolar type II cells, mononuclear cells, and immune and matrix regulatory cells, have potential therapeutic effects on ARDS.

#### Type II alveolar epithelial cells

Like MSCs, AECIIs are recognized progenitor cells in the alveoli. These cells can rapidly proliferate and differentiate into AECIs after epithelial cell injury in the context of ARDS. Moreover, the underlying immunoregulatory and anti-inflammatory properties of AECIIs can restore pulmonary immune and inflammatory homeostasis under pathological conditions in ARDS.^268^ Intratracheal instillation of AECIIs was reported to elevate the levels of surfactant protein A (SPA) and SPC in the alveoli, thus exerting anti-inflammatory effects and consequently maintaining lung homeostasis in rats with ARDS. In addition, the anti-inflammatory effect of AECIIs was also demonstrated by their regulation of alveolar macrophage (AM) polarization, which is associated with a decreased AM1/AM2 ratio and alleviation of pulmonary inflammation.^268^ AECII treatment is comparable to MSC therapy. In rats with LPS-induced ALI, attenuated pulmonary inflammation has been observed after both MSC treatment and AECII treatment, with diminished expression of proinflammatory cytokines, fewer apoptotic cells in lung tissue, and restored pulmonary architecture in the two groups.^269^ All these results from preclinical models of ALI/ARDS have revealed the protective effects of AECII treatment and provided a potential therapeutic direction for ARDS treatment. However, there is still a paucity of data related to AECII treatment for patients with ARDS in the clinic.

#### Immunity- and matrix-regulatory cells

In a preclinical mouse model of ALI, Jun et al. generated immunity- and matrix-regulatory cells (IMRCs), which are derived from human embryonic stem cells (hESCs).^270^ Because of their high expression of immunomodulatory and matrix-regulatory genes, IMRCs exhibit superior immunomodulatory and antifibrotic potency under pathological conditions. Compared with traditional stem cell therapy and pirfenidone injections, intravenous administration of IMRCs to mice with ALI significantly improved lung tissue repair and exerted antifibrosis effects in a dose-dependent manner. Thus, hESC-derived IMRCs are expected to be an alternative cell therapy for ARDS treatment. However, the safety and efficacy of these treatments need to be evaluated in other studies.

### Cell component therapy for ARDS patients

#### Extracellular vesicle-based therapy for ARDS patients

**Fig. 5:**
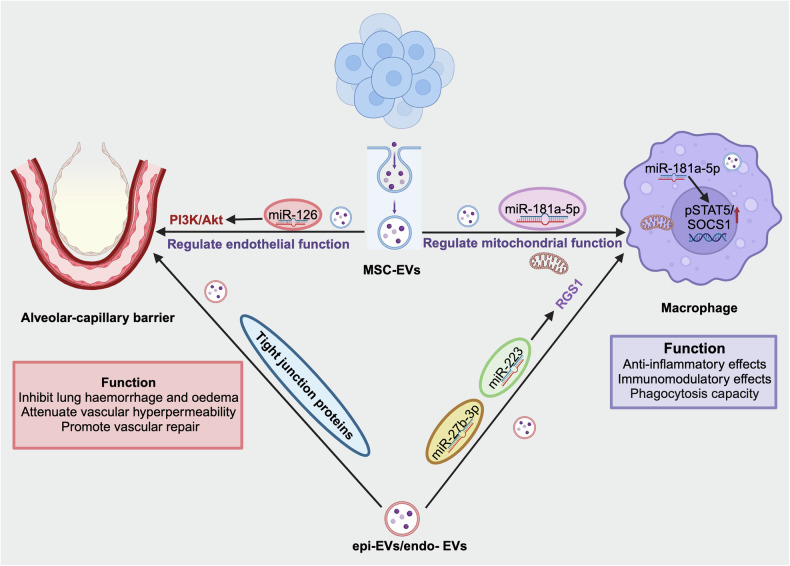
**Extracellular vesicle-based therapy in ARDS**. EVs secreted by MSCs or other cells primarily exert their effects by cargos such as miRNAs and mitochondria. These contents can alleviate alveolar-capillary barrier damage, and regulate macrophage function, thereby achieving anti-inflammatory and immune regulatory functions. *EVs extracellular vesicles, MSC mesenchymal stromal cell, epi-EVs epithelium-derived extracellular vesicles, endo-EVs endothelium-derived extracellular vesicles

Although stem cells have therapeutic effects, they also have several disadvantages and adverse effects. Several cell products derived from stem cells, especially from MSCs, have been explored for efficacy and safety in the context of ARDS treatment. Among these cell products, stem cell-derived EVs play a central role in lung injury repair and respiratory function restoration and have advantages over MSC therapy, including low immunogenicity, prolonged in vivo stability, high delivery efficiency, and a low risk of inducing iatrogenic tumor formation.^271^ An increased number of microvesicles (MVs) has been observed in both ARDS patients and rodents with ALI, which indicates the critical role of MVs, especially EVs, in the initiation, development, and progression of ARDS. Endothelial cell-derived microparticles and leukocyte microparticles in the circulation have been identified as prognostic markers of ARDS development in clinical patients with sepsis.^272,273^ In a prospective trial, EV-derived or EV-encapsulated miRNAs were reported as biomarkers to distinguish ARDS patients with or without COVID-19, which might provide a new direction for treating CARDS,^274^ shown in Fig. 5.

Nowadays, EVs, including MSC-derived EVs and EVs derived from other cells, which attenuate ALI mainly by regulating cellular activities such as apoptosis and autophagy,^275^ mitochondrial function, and alveolar epithelial-capillary barrier integrity in pulmonary tissues, have been widely applied in animal models of ARDS/ALI. Macrophage dysfunction appears to be essential in the pathogenesis of ARDS, and targeting macrophage dysfunction might thus be a therapeutic direction for treating ARDS. MSCs-EVs or MSCs-MVs promote macrophage phagocytosis of pathogens in lung tissue, thus attenuating pulmonary inflammation and lung injury in both mouse and rat ALI models.^276,277^ Further investigation demonstrated that miR-181a-5p enveloped in MSC-EVs could upregulate pSTAT5 and SOCS1 expression in alveolar macrophages, exerting anti-inflammatory and immunomodulatory effects in the context of ARDS.^278^ Additionally, MSCs-EVs modulate macrophage phenotypes and enhance polarization to the anti-inflammatory and highly phagocytic phenotype, which mainly depends on improved macrophage oxidative phosphorylation in mitochondria due to EV-mediated mitochondrial transfer.^279^ MSCs-EVs can regulate alveolar macrophage autophagy, enhance homeostasis in alveolar macrophages, and alleviate mitochondrial dysfunction-mediated barrier damage, thus reducing lung injury during ARDS via EV-mediated mitochondrial transfer.^280–283^ AD-MSC-EV administration also regulates endothelial functions in pulmonary tissue, with inhibition of lung hemorrhage and edema, attenuation of vascular hyperpermeability, and promotion of vascular repair in mice with ALI^284^; these effects rely on PI3K/Akt activation by EV-encapsulated miR-126 in endothelial cells.^285^ These findings indicate that MSCs-EVs exert powerful effects during ARDS therapy.

Interestingly, the anti-inflammatory and immunomodulatory properties of alveolar macrophages can also be regulated by epithelium- and endothelium-derived EVs (epi-EVs or endo-EVs) that target RGS1 activation and mediate the intracellular Ca^2+^ response in the context of ARDS, mainly through miR-27b-3p and miR-223, respectively.^286^ The intratracheal administration of endothelial progenitor cell-derived EVs (EPC-EVs) also reduces vascular permeability and inflammation via the overexpression of tight junction proteins, which suggests that EPC-EVs have endothelial protective effects in ARDS.^287^ However, contrary to previous results, pulmonary epithelial cell-derived EVs were reported to activate alveolar macrophages and aggravate pulmonary inflammation during ARDS because they are hyperoxia-induced, enriched in caspase-3, and thus proinflammatory.^288^ Thus, more research is needed to elucidate the potential prognostic and therapeutic effects of epi-EVs in ARDS.

#### Plasma therapy and plasma protein-based therapy

In recent decades, plasma therapy and plasma protein-based therapies have been widely used to treat patients with severe CARDS. However, convalescent plasma therapy (CPT) is controversial since previous research has shown conflicting outcomes in terms of the benefit or lack of benefit of this treatment. Recently, two randomized, double-blind, multicentre, phase 2 and phase 3 clinical trials have been conducted to investigate the efficacy and safety of CPT for severe influenza infection.^289,290^ The study results did not suggest that CPT has superior therapeutic effects compared to nonimmune plasma therapy, while convalescent plasma treatment seems safe and well tolerated. Shen and colleagues reported decreased viral loads and increased neutralizing antibody levels in CARDS patients’ plasma after CPT, and the patients’ symptoms and outcomes improved as well.^291^ Inspired by these case series, two clinical trials have been performed, with conflicting results. In the first study, when CARDS patients were treated with plasma, clinical improvements were observed, including reductions in disease severity, length of ICU stay, and the need for mechanical ventilation and better oxygenation,^292^ which indicates the efficacy of CPT in patients with CARDS. In contrast, in the second study, although no adverse effects were observed in the CPT group, significant improvements in clinical outcomes were not detected after CPT.^293^ The discrepancy between these two studies might be related to the use of different sources of CP resources, leading to a potential difference in the antibody level of the donors.

Further studies have investigated the presence of immunoglobulins and other monoclonal antibodies in the plasma to eliminate confounding factors. Intravenous immunoglobulins (IVIGs) can improve clinical outcomes in patients with CARDS by reducing mortality, length of ICU stay, and duration of mechanical ventilation.^294–297^ However, a multicentre, double-blind, placebo-controlled trial confirmed that IVIGs do not improve primary clinical outcomes, with a similar number of ventilation-free days on day 28 with or without IVIGs.^298^ In addition, IVIGs seem to increase the incidence of several serious adverse events in ARDS patients, such as septic shock, acute kidney injury, and bacterial pneumonia. There are two possible reasons for the differences in the results of these studies. First, the beneficial effects of IVIGs have been reported in retrospective observational studies, which are more prone to selection bias. Second, a nonbeneficial or detrimental impact of IVIGs has been demonstrated in more severe ARDS patients, which might cause poor clinical outcomes, even without IVIGs.

## Targeted therapy

Targeted therapy is a treatment strategy in which specific molecules associated with a disease are targeted to regulate pathological and physiological processes and achieve therapeutic goals. Compared to traditional broad-spectrum methods, targeted therapy is more effective and has fewer side effects.^299^ Targeted therapy can be achieved through the use of antibody drugs,^300^ small molecule targeted drugs,^301^ gene therapy,^302^ or RNA interference technology.^303^ It is a highly personalized treatment approach that requires a deep understanding of the molecular mechanisms of the disease and has a wide range of applications thanks to the development of genomics and proteomics techniques.

### Treatments targeting the immune system

#### Advantages

Since consensus on the definition of ARDS was reached in 1988, scientists have been trying to find specific therapeutic drugs for ARDS. However, many potential drugs have failed in clinical trials, mainly due to the significant clinical and biological heterogeneity of ARDS^147^; for example, the “low inflammatory” subtype and “hyperinflammatory” subtype,^147,304^ have different therapeutic characteristics. Individualized treatment plans can be selected for patients receiving targeted therapy based on phenotype and molecular pathology characteristics. This approach can achieve site-specific drug delivery and has good potential for reducing off-target effects and unnecessary toxicity and enhancing the therapeutic effects of drugs.^301^ Immune dysregulation is a vital pathogenic mechanism of ARDS that is characterized by sustained and excessive activation of the inflammatory response, abnormal elevation of cytokine levels, and suppression of anti-inflammatory responses. The immune phenotypes of different ARDS patients may be significantly different,^305^ so treatments targeting the immune system have become a vital approach for ARDS patients.

#### Targeted immunomodulatory therapies

##### Targeted immunomodulatory therapies for non-COVID-19-induced ARDS

1. Neutrophils
  Neutrophils are the primary immune cells that cause the inflammatory response in ARDS. Sivelestat, a neutrophil elastase inhibitor, has been approved in Japan for the treatment of ALI in patients with systemic inflammatory response syndrome. A phase IV clinical trial recruited 177 patients in the Sivelestat group and 15 patients in the control group. The results showed that compared to those in the control group, the adjusted extubation rate and ICU discharge rate were significantly greater in the Sivelestat group, with a considerably higher adjusted 180-day survival rate (71.8% vs. 56.3%).^306^ A retrospective study also demonstrated that Sivelestat had beneficial effects on sepsis-related ARDS.^307^ In addition, a meta-analysis including six RCTs with a total of 804 ALI/ARDS patients showed that sivelestat may increase PaO2/PaCO2 but has little or no effect on the 28-30-day mortality rate, ventilator days, or duration of ICU stay.^308^ However, larger sample sizes are needed to verify the effectiveness of Sivelestat in the treatment of ARDS.
2. TNF-α
  TNF-α is an essential inflammatory factor involved in ARDS that can induce cell death and inflammatory responses after binding to TNFR 1 and TNFR 2 on the cell membrane. In the context of acute injury, selective inhibition of TNFR1 may reduce the adverse effects associated with TNF-α while preserving or enhancing the protective effects of TNFR2. Based on this mechanism of action, the inhalation of GSK 1995057, a selective TNFR1 antagonist, is considered a possible therapeutic approach for preventing ARDS.^309^ A randomized controlled clinical trial involving 37 healthy individuals showed that the inhalation of GSK-1995057 can prevent the increase in neutrophil counts, inflammatory cytokine levels, and endothelial cell injury observed in healthy individuals exposed to inhaled endotoxin.^310^
3. GM-CSF
  Granulocyte-macrophage colony-stimulating factor (GM-CSF) is a multifunctional cytokine that has potent effects on cell activation and differentiation and can enhance the antibacterial functions of bone marrow cells (macrophages, dendritic cells, and PMNs) and alveolar macrophages.^311^ Although a multicentre phase II trial showed that GM-CSF treatment in ARDS patients did not change the duration of mechanical ventilation compared to that of patients in the placebo group, it did reduce nonpulmonary organ failure and mortality (absolute reduction of 6%, relative reduction of 26%).^312^ Consistently, in phase II trials of sepsis patients with respiratory dysfunction, treatment with GM-CSF improved respiratory function.^313^
4. Vascular leakage
  Vascular leakage is one of the main pathophysiological mechanisms of ARDS, and Abl family kinases are critical mediators affecting vascular permeability. Imatinib (STI-571, Gleevec) is a prototype Abl kinase inhibitor that has been shown to reduce the production of proinflammatory cytokines (TNFα, IL-8, and IL-1β) and alleviate lung injury in a mouse model of ALI.^314^ A series of case reports suggest that Abl kinase inhibitors may reduce vascular permeability in specific patients. Overbeek et al. described a patient with pulmonary arterial hypertension and suspected pulmonary veno-occlusive disease who showed improved dyspnea and reduced pulmonary edema within 24 hours of starting imatinib treatment.^315^ Carnevale et al. reported a patient with bleomycin-induced pneumonia who showed rapid improvements in respiratory status and radiographic findings after imatinib treatment.^316^ Aman et al. reported that a patient with idiopathic vascular leakage experienced a nearly complete recovery after starting imatinib treatment, including resolution of dyspnea and cough and normalization of vascular leakage-related parameters.^317^ Therefore, Abl kinase inhibitors with different specificities may help alleviate vascular inflammation and leakage and may become targeted drugs for the treatment of ARDS.
5. Antibiotics
  It has been reported that some antibiotics, including macrolides and tetracyclines, have pleiotropic immunomodulatory effects in addition to antibacterial effects.^318^ In addition, increasing evidence supports the beneficial effects of these antibiotics on acute inflammatory processes such as pneumonia and ARDS.^319^ A recent prospective randomized controlled trial demonstrated that the addition of clarithromycin to the standard of care enhances early clinical response and attenuates inflammation in patients with community-acquired pneumonia, and these changes were associated with changes in the immune response.^320^ Sauer A’s review systematically described preclinical and clinical studies on the immunomodulatory effects of antibiotics in ARDS and the underlying mechanisms of their immunomodulatory effects. That review concluded that this unique antibiotic is a potent modulator of the innate immune response and may improve immune dysregulation in ARDS. This effect depends on the administration time, frequency, dose, and degree of lung injury, but further studies are needed to resolve contradictory results from preclinical and clinical results.^321^
6. KGF
  Keratinocyte Growth Factor (KGF) enhanced physiological outcomes and markers of alveolar epithelial cell function in various animal models of ARDS/ALI. In a human in vivo short-term model of acute lung injury and ARDS, KGF (palifermin) pretreatment increased bronchoalveolar lavage concentrations of SP-D, a marker of type II cell proliferation, indicating its promotion of epithelial cell survival, supporting its potential benefits in ARDS.^322^ However, a randomized Phase 2 clinical trial of KGF in patients with ARDS revealed that VFD over 28 days was reduced, but mortality was higher at 28 days in patients treated with KGF, suggesting that it might exacerbate clinical outcomes in ARDS.^323^ Therefore, KGF might not be used to treat patients with ARDS.
7. Tie-2 antagonist

The activation of Tie2 by angiopoietin-1 (Ang-1) leads to a reduction in inflammation and endothelial permeability in sepsis.^324^ Vasculotide (VT) is a polyethylene glycol-clustered Tie2-binding peptide that mimics the actions of Ang-1. VT has been shown to promote pulmonary endothelial stability and reduce lung permeability in various models of pneumococcal pneumonia. Treatment with the angiopoietin-1 mimetic vasculotide has been found to reduce pulmonary vascular leakage and preserve microcirculatory perfusion during cardiopulmonary bypass (CPB) in a rat model.^325^ Interestingly, combining VT with ampicillin significantly reduced pulmonary hyperpermeability, histological lung damage, and edema formation, without altering the immune response or bacterial burden.^326^

##### Targeted immunomodulatory therapies for CARDS

Upon SARS-CoV-2 invasion, the release of cytokines and chemokines may cause a “cytokine storm,” leading to ARDS and multiple organ dysfunction syndrome (MODS).^327^ Proinflammatory cytokines, monocytes, and neutrophils may be critical players in the development of the cytokine storm in patients with severe CARDS. Therefore, blocking cytokines may be a crucial strategy for CARDS treatment. Some drugs targeting immunopathology, such as interferons, thymosin, and related cytokine inhibitors, have shown therapeutic effects in patients with severe CARDS.

1. Thymosin
  Thymosin is a physiologically active small peptide extracted from the thymus of humans and animals^328^ that can promote thymocyte differentiation and maturation and increase the number and activity of T cells.^329^ In patients with COVID-19, thymosin may limit disease progression by stimulating lymphocyte production, particularly in patients with reduced lymphocyte counts. A retrospective cohort study including 334 COVID-19 patients showed that the use of thymosin α1 significantly reduced the 28-day mortality rate and attenuated ALI in critically ill patients.^330^ However, another multicentre retrospective study including 771 patients did not find a correlation between thymosin α1 use and a decreased mortality rate among critically ill COVID-19 patients.^331^ This inconsistency may be due to differences in study subjects, COVID-19 severity, sex ratios, and thymosin dosage and timing. Thus, further clinical trials are needed to verify the effectiveness of thymosin α1.
2. GM-CSF
  GM-CSF is believed to be a vital mediator of excessive inflammation in patients with COVID-19.^332^ Several clinical trials have investigated the use of monoclonal antibodies to treat COVID-19 by modulating the immune response via GM-CSF or its receptor (GM-CSFR) to reduce the inflammatory signaling activated by the GM-CSF axis. Among the investigated antibodies, lenzilumab (a recombinant monoclonal antibody against GM-CSF)^333^ and mavrilimumab (a monoclonal antibody against GM-CSFRα)^334^ have been shown in small prospective cohort studies to be associated with reduced mortality and improved systemic hyperinflammation in patients with severe COVID-19. However, a randomized, double-blind, placebo-controlled trial of Gimsilumab, a monoclonal antibody against GM-CSF, in 225 COVID-19 pneumonia patients revealed no significant changes in mortality, non-IMV-free survival, non-IMV duration, time to hospital discharge, or systemic inflammation.^335^ Therefore, the efficacy of anti-GM-CSF or anti-GM-CSFR therapy for CARDS patients remains unclear. Additionally, the recombinant human granulocyte colony-stimulating factor (rhG-CSF) could reduce the occurrence of CARDS.^336^ However, rhG-CSF fails to alleviate the severity in patients with COVID-19, and the underlying therapeutic effects and potential mechanisms remain ambiguous in CARDS.
3. Interleukin
  IL-1β and IL-6 play pivotal roles in cytokine storm-related pathways, mediating pathological processes such as fever, pulmonary inflammation, and fibrosis by binding to Toll-like receptors.^337^ Anakinra, a 17 kDa recombinant, nonglycosylated human IL-1 receptor antagonist.^338^ Blocking IL-1 can serve as an adjunctive therapeutic approach for the management of patients with severe COVID-19. An RCT including 30 COVID-19 patients demonstrated that the anakinra group had a significantly lower need for IMV than did the control group, with significantly shorter hospital stays and no notable increase in infection rates.^339^ Another small-sample retrospective study indicated that high-dose (5 mg/kg intravenous infusion) anakinra significantly improved patient survival and improved the clinical condition, inflammatory factors, and mechanical ventilation requirements of CARDS patients outside the ICU compared to low-dose anakinra (100 mg subcutaneous injection twice daily).^340^ A meta-analysis of 1185 patients with moderate to severe COVID-19 revealed a significant reduction in 28-day mortality with anakinra use.^341^
  Sarilumab is a human monoclonal antibody that directly inhibits the binding of IL-6 to its receptor.^342^ It inhibits both soluble and membrane-bound forms of the IL-6 receptor, potentially suppressing the activation of proinflammatory signals in pulmonary epithelial cells and immune cells.^343^ An open-label, randomized, controlled phase II clinical trial demonstrated the safety and potential efficacy of early use of 400 mg sarilumab in patients with COVID-19 pneumonia and features of systemic inflammation.^344^ In a multicentre phase III clinical trial, 416 COVID-19 pneumonia patients were randomized to receive different doses of sarilumab (200 mg or 400 mg). Although sarilumab did not demonstrate differential efficacy overall, there was a survival difference between the sarilumab 400 mg group and the placebo group on day 29 in critically ill patients (88% vs. 79%), suggesting that appropriate IL-6-targeted immunomodulatory treatment in critically ill COVID-19 patients may improve patient prognosis.^345^
4. Complement system
  COVID-19 is characterized by severe pulmonary inflammation and activation of coagulation, leading to adverse disease outcomes associated with the activation of the complement system, particularly the C5A-C5AR axis.^68^ Blocking the C5A-C5AR1 axis can limit the infiltration of myeloid cells into damaged organs, preventing excessive pulmonary inflammation and endothelial cell dysfunction.^346^ A multicentre, double-blind, randomized phase 3 trial, which included 368 patients with CARDS, showed a significant reduction in the all-cause mortality rate at 28 days after anti-C5a antibody (vilobelimab) therapy.^347^ Ruxolitinib, an inhibitor of Janus kinases 1 and 2, which play a crucial role in inflammation, has shown promise in treating COVID-19 patients with severe systemic inflammation.^348,349^ In a nonrandomized prospective phase II multicentre study, ruxolitinib was used for targeted inhibition of Janus kinase-mediated cytokine signaling and was found to improve the prognosis of patients with COVID-19-induced ARDS when given immediately after disease deterioration.^350^ In summary, vilobelimab and ruxolitinib have shown potential as treatments for treating CARDS, but more clinical trials and animal experiments are needed to develop effective complement inhibitors for COVID-19 treatment.
5. NETs
  The ability of neutrophils to form NETs may contribute to organ damage and mortality in COVID-19 patients, as supported by autopsy findings; this may be related to the oxidative damage caused by the release of extracellular DNA.^351,352^ Dornase Alfa is a recombinant human deoxyribonuclease I that degrades extracellular DNA and has mucolytic properties.^351^ A pilot, nonrandomized, case‒controlled clinical trial revealed that the Dornase Alfa group exhibited a significant improvement in the PaO2/FiO2 ratio on day two and a substantial improvement in static lung compliance on days 3-5 compared to the control group. Furthermore, Dornase Alfa treatment improved oxygenation in the BALF and reduced DNA release via the formation of myeloperoxidase (MPO) complexes.^353^ This finding suggested that Dornase Alfa may have therapeutic potential in mitigating the severity of CARDS by reducing NET-mediated lung injury.
6. TNFSF14
  Lymphocyte-inducing protein (LIGHT or TNFSF14) is a cytokine that competes with glycoprotein D to prevent viruses from entering T cells and plays a critical role in creating a network of cytokines and receptors that regulate the host defence system and the communication systems that control immune responses.^354^ The efficacy and safety of CERC-002, a human neutralizing antibody against TNFSF14, for treating COVID-19-induced ARDS patients were assessed. Among 83 patients, those who received CERC-002 treatment had a significantly higher rate of survival free of respiratory failure (83.9%) than did those who received a placebo (64.5%) at day 28, as well as lower 28-day and 60-day mortality rates.^355^ These findings suggest that CERC-002 may be a promising treatment option for patients with CARDS.
7. NLRP3

One of the essential functions of the innate immune system is to recognize pathogens by triggering the NLRP3 inflammasome.^356^ DFV890 is a novel, potent, and selective oral NLRP3 inhibitor.^357^ Madurka et al. conducted a multicentre study including 143 patients with COVID-19 pneumonia who were randomized to the DFV890 + standard-of-care (SoC) group or the SoC-alone group. The results showed that the DFV890 group achieved better outcomes than the SoC group, with clinical improvement ≥1 grade, earlier viral clearance, earlier survival without mechanical ventilation, and lower mortality.^358^ However, further studies are needed to confirm its safety and efficacy in larger patient populations and to identify the optimal dosing and treatment regimens.

Targeted immunotherapy has emerged as a promising approach for treating ARDS; however, several challenges must be overcome to maximize its therapeutic potential. The heterogeneity of ARDS presents a significant challenge in identifying specific therapeutic targets that can benefit all patients uniformly. Moreover, the timing of immunotherapy is crucial, as initiating treatment either too early or too late may have detrimental effects on the clinical outcome. Additionally, the potential for off-target effects, including immune suppression or undesired effects on nonpathogenic cells, may limit the safety of immunotherapy. Cost is another critical factor that may limit the availability and accessibility of targeted immunotherapy. In this regard, personalized medicine strategies may represent a future direction for targeted immunotherapy. These strategies aim to develop patient-specific treatments that consider individual factors such as genetics, comorbidities, and disease severity. The use of biomarkers and advanced imaging techniques may also aid in identifying appropriate targets and monitoring treatment response. Furthermore, combination therapies that target multiple pathways or utilize different modalities, such as gene therapy or cell-based therapies, may provide a more comprehensive and effective approach to improve outcomes in ARDS patients.

### Other targeted therapies

In addition to targeted immunomodulatory therapies, there are many other potential targeted drugs for ARDS treatment. NLRP3, NF-κB, STAT, Nrf2, TNFR1, and SIRT1 are the most common targets of inhibition, and multiple miRNAs, which are key regulatory factors in signaling pathways, have also been shown to be beneficial in animal models of ARDS. Notably, natural products and traditional Chinese medicines have application prospects in ARDS treatment. However, most of these drugs are still in the preclinical stage, and there is a lack of information on their safety, ideal concentrations, administration methods, etc., in ARDS patients. More details are shown in Table 5.^359–445^

**Table 5:** Other targeted drugs

Targets of inhibition	Type of drugs	Name of drugs	The inflammatory factors affected	Methods for inducing the model	Ref
**NLRP3**	Natural products or traditional Chinese medicine	Arglabin	IL-1β, IL-18	LPS	^359^
		Borago officinalis	IL-1, IL-18	LPS	^360^
		Curcumin	IL-1β, TNF-α	LPS	^361^
		Tectoridin	IL-6, IL-18, IL-1β	LPS	^362^
		Emodin	TNF-α, IL-6, IL-1β	LPS	^363^
	Transcription factors	Heat shock factor 1	IL-1β, IL-18	LPS	^364^
	Hormones	Melatonin	TNF-α, IL-3β, IL-1β	LPS	^365^
	4-benzene-indol derivative	1,2-diol	IL-1β, TNF-α	LPS	^366^
	Cysteine derivatives	Fudosteine	IL-6, KC, IL-1β, IL-18	LPS	^367^
	Nanoparticles	Nano-chemically Modified Tetracycline-3	TNF-α, IL-1β, IL-6, IL-18	LPS	^368^
	Hypoglycemic drugs	Glibenclamide	IL-1β, IL-18	LPS	^369^
	Mitochondrial membrane proteins	FUNDC1	TNF-α, IL-1β, IL-6, IL-10	LPS	^370^
	Anti-allergic agent	Tranilast	IL-13, IL-33, IL-5	LPS	^371^
	NLRP3 inhibitor	MCC950	IL-1β, IL-8, TGF-β1, MMP-9	LPS	^372^
	Alpha-2 adrenoceptor agonists	Dexmedetomidine	IL-1β, IL-18, IL-6, TNF-α	THSR	^373^
	Gene silencing	NLRC4	IL-1β, TNF-α, IL-6	CLP	^374^
	Gene knockout	PIM2	IL-1β	LPS	^375^
	Pyridinone	Pirfenidone	IL-1β, TGF-β1	LPS	^376^
**NF-κB**	Cold shock protein	RBM3	IL-1β	LPS	^377^
	Neutrophil elastase inhibitor	Sivelestat sodium	L-1β, IL-8, TNF-α	TNF-α	^378^
	Alkaloid	Milonine	IL-1β, IL-6, TNF-α	LPS	^379^
	Organosulfur compound	Allyl methyl trisulfide	IL-6, IL-1β, TNF-a	LPS	^380^
	Antiplatelet agent	Aspirin	TNF-α, IL-1β, and IL-4	LPS	^381^
	Gene silencing	OLFM4	IL-6, CXCL-1, LCN2	CLP	^382^
	Natural products or traditional Chinese medicine	Andrographolide Suifosalt Injection	IL-1β, MPO	LPS	^383^
		Dehydrozingerone	IL-6, IL-1β, TNF-α	LPS	^384^
		Berberine	IL-1β, TNF-α	LPS	^385^
		Emodin	IL-8, IL-1β, TNF-α	LPS	^386^
		Gentiopicroside	IL-1β, IL-6, TNF-α	LPS	^387^
		Nimbolide	TNF-α	LPS	^388^
		Euphorbia factor	IL-1 β, IL-6, TNF- α, IL-8	LPS	^389^
		Gymnemic Extract	IL-6, IL-1β	LPS	^390^
		Osthole	IL-6, TNF-α	LPS	^391^
		Palrnatine	IL-‍1β	LPS	^392^
		Garcinia and gallic acid	TNFα, IL-6, IL-1β, IL-8	SARS-CoV-2 spike glycoprotein S1	^393^
**STAT**	Probiotics	Lactobacillus rhamnosus	IL-10, IL-12, IL-17, IL-18, IL-22, IL-23, IL-27, IFN-γ	LPS	^394^
	Endogenously pro-resolution lipid mediator	MCTR1	IL-4, IL-13, IL-1β	LPS	^395^
	pulmonary surfactant	SPC	IL-5, IL-9, IL-12, IL-13,	SPC-KO	^396^
	STAT3 inhibitor	LLL3	IL-6β、IL-2、TNF-α	LPS	^397^
	Macrolides	Clarithromycin	IFN-γ	LPS	^398^
	Selective antagonist of the AT1 receptor	Losartan	TNF-a, IL-1b, IL-8	Seawater inhalation	^399^
**MicroRNA**	Targeting TNFR1	MiR-29a-3p	IL-1β, IL-6, TNF-α	LPS	^400^
	Targeting interleukin receptors	MiR-155-5p	IL-17RB, IL-18R1, IL-22RA2	LPS	^401^
	Targeting IL1RN	MiR-122-5p	TNF-α, IL-1β, IL-6	LPS	^402^
	Targeting HMGB1	MiR-574-5p	IL-6, IL-1β, TNF-α	LPS	^403^
	Targeting RUNX2	MiR-30a-5p	IL-6β, IL-5, IL-4, IL-9, TNF-α	LPS	^404^
	Targeting ROCK1	MiR-539-5p	IL-1β, IL-6	LPS	^405^
	Targeting CDK8	MiR-297	IL-1β, IL-6, TNF-α	LPS	^406^
	Targeting TREM-1	MiR-155	IL-1β, IL-6, TNF-α	LPS	^407^
	Targeting CDKN1B	MiR-877-5p	IL-1β, IL-6, TNF-α	LPS	^408^
	Targeting P65	MiR-124-3p	IL-1β, IL-6 and TNF-α	LPS	^409^
	Targeting Klf2	MiR-34a	IL-1β	LPS	^410^
	Targeting IRAK1	MiR-146a	IL-6, IL-8, and TNFα	Mycin	^411^
**Nrf2**	Macromolecular transmembrane glycoproteins	Mucin 1	TNF-α, IL-1β, IL-6, IL-10	LPS	^412^
	mRNA binding protein	AUF1	TNF-α, IL-1β, and IL-6	CLP	^413^
	Anti-hypertensive agent	Azilsartan	IL-1β, MCP-1, IL-8	LPS	^414^
	Nrf2 agonist	Sulforaphane	TNF-α, IL-6, MCP-1	HS/R	^415^
	Melatonin Receptor Agonist	Ramelteon	TNF-α, IL-1β, IL-6	LPS	^416^
	Tyrosine kinase inhibitor	Dasatinib	IL-6, TNF-α, IL-10	LPS	^417^
	Mitochondria-targeted antioxidant	MitoQ	IL-6, TNF-α	LPS	^418^
	EphA2 antagonist	EphA2 antagonism	TNF-α, IL-6	LPS	^419^
	Natural products or traditional Chinese medicine	Isorhapontigenin	IL-1β, IL-6, TNF-α	LPS	^420^
		TADIOS	IL-6, IL-1β	LPS	^421^
		Panaxydol	TNF-α, IL-1β, IL-6	LPS	^422^
		Vincamine	TNF-α, COX-2	LPS	^423^
		Qiwei Putao powder	IL-1β, TNF-α	LPS	^424^
**TNFR1**	Fungal metabolite	Allantopyrone A	RIP1, NF-κB	TNF-α	^425^
	Fragment of IgG	Domain antibody (dAb™)	IL-6, CXCL1	Acid inhalation	^426^
	Endothelin receptor antagonist	HJP272	Neutrophils, Macrophages	LPS	^427^
	TNF-α inhibitor	Etanercept	TNF-α, IL-1β	Zymosan	^428^
**SIRT1**	α2-adrenergic receptor agonists	Dexmedetomidine	Foxp3, IL-10, TNF-α, IL-6, IL-17	CLP	^429^
	Hormone	Melatonin	TNF-α, IL-6, IL-10	CLP	^430^
	Synthesized raceme of L-3-n-butylphthalide	DL-3-n-butylphthalide	TNF-α, IL-6	LPS	^431^
	Natural products or traditional Chinese medicine	Curcumin	MDA, TNF-α	HS/R	^432^
		Tanshinone IIA	TNF-a, IL-1b, IL-6	LPS	^433^
		Hydroxytyrosol	TNF-α, IL-1β, IL-6, IL-10, and MCP-1	LPS	^434^
		Resveratrol	TNF-α, IL-6, HMGB1	LPS	^435^
**NET**	Phenolic diterpene	Carnosic acid	CD11b, MPO	LPS	^436^
	Selective inhibitors of nuclear export	Selinexor	TGF-β, TNF-α, IL-8	LPS	^437^
	Endogenous mediators	Lipoxin A4	TNF-α, IL-1β	LPS	^438^
	Glutathione reductase	Glutamine	IL-1β、IL-10, IFN-γ	LPS	^439^
	Phospholipase and tyrosine kinase inhibitors	β-Nitrostyrene Derivatives	TNF-α, IL-6	LPS	^440^
	Natural products and herbal extracts	Xuebijing injection	CSF-3, CXCL-2, CXCR-2	CLP	^441^
		Salvianolic acid A	TNF-α, IL-6, IL-1β	LPS	^442^
		Bletinib	MPO, IL-1β	LPS	^443^
		Re-Du-Ning injection	IL-1β, IL-6, TNF-α	LPS	^444^
		Polydatin	IL-6, IL-1β, TNF-α, MCP-1	LPS	^445^

Many targeted drugs of ARDS have shown significant benefits in animal experiments, particularly in improving inflammation, but more data are needed to clarify their efficacy and safety

*NLRP3 NOD-, LRR- and pyrin domain-containing 3, *IL* interleukin, *LPS* lipopolysaccharide, *TNF-α* tumor necrosis factor alpha, *KC* keratinocyte-derived chemokine, *FUNDC1* FUN14 domain-containing protein 1, an integral mitochondrial outer-membrane protein, *MCC950* an NLRP3 inflammasome inhibitor, *MMP* matrix metalloproteinase, *THSR* blunt chest trauma and hemorrhagic shock and resuscitation, *NLRC4* NLR family CARD domain-containing protein 4, *CLP* cecal ligation and puncture, *PIM2* a member of the serine/threonine kinases family, *TGF* transforming growth factor, *NF-κB* nuclear factor kappa B, *RBM3* cold-shock protein RNA binding motif 3, *CXCL* C-X-C motif ligand, *LCN* lipocalin, *MPO* myeloperoxidase, *STAT* signal transducer and activator of transcription, *IFN* interferon, *MCTR* maresin conjugates in tissue regeneration, *SPC* surfactant protein C, *LLL* a small molecule STAT3 inhibitor, *HMGB* high mobility group box, *RUNX* runt-related transcription factor, *ROCK IL1RN* interleukin-1 receptor antagonist, *ROCK* Rho-kinase, *CDK* cyclin dependent kinase, *TERM* triggering receptor expressed on myeloid cells, *CDKN* cyclin-dependent kinase inhibitor, *Klf* kruppel-like factor, *IRAK* interleukin-1 receptor-associated kinase, *Nrf2* nuclear factor-E2-related factor 2, *AUF* AU-binding factor, *MCP* monocyte chemoattractant protein, *HS/R* Hemorrhagic shock/resuscitation, *Eph* Ephrin receptors, *TADIOS* an herbal formulation prepared from a mixture of Taraxacum officinale, *COX* cyclooxygenase, *TNFR* TNF receptor, *RIP* receptor-interacting protein, *HJP272* a novel endothelin receptor A antagonist, *SIRT* sirtuins, *MDA* malondialdehyde, *NET* neutrophil extracellular trap, *CD* cluster of differentiation, *CSF* colony-stimulating factors

## Personalized therapy for ARDS

Significant differences in genetic, biological, and environmental factors among ARDS patients, including differences in etiological profiles, immune profiles, and inflammation profiles, can lead to differences in responsiveness to treatment.^446^ Thus, evidence suggests that personalized medicine approaches should be used for patients with different ARDS subphenotypes. Before we achieve personalized medicine strategies for ARDS, we must determine the exact phenotypes of ARDS patients. As we discussed above, the selected treatments must be targeted and effective for the patient’s specific ARDS phenotype.^447^

### Personalized medicine

To date, personalized medicine for ARDS patients has mainly depended on the microenvironment in pulmonary tissue and the whole body, clinical features, ventilator parameters, etc. However, ARDS clinical phenotypes provide a new therapeutic direction for personalized medicine. Several researchers have demonstrated that therapeutic effects can vary according to the subphenotype of ARDS, as discussed above. For example, patients with hyperinflammatory ARDS may benefit from treatments such as simvastatin, corticosteroids, and MSCs, while other patients may experience no benefit or even detrimental effects.^147,448,449^ Therefore, the investigation of personalized medicine approaches selected according to the clinical phenotypes of ARDS would be valuable in future studies.

### Personalized mechanical ventilation

Personalized mechanical ventilation has recently emerged as a management approach for ARDS patients. In Jean’s study, the authors compared mortality and other clinical outcomes among several subgroups of ARDS patients, which were grouped according to lung morphology.^450^ The results showed that correct adjustment of the ventilator strategy according to lung morphology decreased mortality compared to that observed for unclassified patients and patients receiving a misaligned ventilator strategy, suggesting that personalized mechanical ventilation selected according to an accurate classification of lung morphology may be a potential therapeutic direction for ARDS patients. Furthermore, ventilator parameter-focused strategies might be beneficial for treating ARDS. Tidal volume was previously thought to be the primary factor involved in developing ventilator strategies. However, a recent study revealed that driving pressure, which is determined by tidal volume and lung elastance, is more important in the development of ventilator strategies,^451^ which indicates that driving pressure-targeted lung-protective ventilation strategies are useful for treating ARDS.

### Organ function-dependent personalized strategy

Organ function is also important in the development of personalized management strategies for ARDS patients. It has been reported that PEEP, especially esophageal pressure-guided PEEP (Pes-guided PEEP), is associated with patient mortality in ARDS patients with variations in organ function.^452^ Pes-guided PEEP is associated with better clinical outcomes in patients with higher APACHE-II scores, which indicate severe multiorgan dysfunction in ARDS patients. In contrast, Pes-guided PEEP is associated with lower mortality among patients with better organ function. All the above results suggest that organ function-dependent PEEP strategies might be an alternative approach for personalized mechanical ventilation in ARDS patients. Organ function can also be instructive for personalized fluid strategies in ARDS patients. On the basis of their clinical symptoms and organ function parameters, ARDS patients can be divided into several phenotypes, including phenotype 2, which is characterized by more inflammation and less organ dysfunction, and phenotype 3, which is characterized by severe renal dysfunction and acidosis.^125^ Interestingly, the fluid-conservation strategy has beneficial effects on mortality in patients with phenotype 3 disease but detrimental effects on mortality in patients with phenotype 3 disease, indicating the potential of personalized fluid management strategies for patients with ARDS.

## Other alternative therapy

Other treatments for ARDS, such as inhaled carbon monoxide, vitamins and other drugs have also been investigated. Recently, vitamins have emerged as agents for managing ARDS, as vitamin D appears to attenuate lung damage caused by CARDSs and ALI, according to preclinical studies.^453^ However, continuous infusion of vitamin C did not improve multiorgan function or attenuate serum markers of inflammation and vascular injury in patients with sepsis-related ARDS.^454^ This difference may have several causes. First, differences across species may have led to different results. Second, different phenotypes of ARDS have unique pathogenic mechanisms and clinical features, resulting in different responses to vitamin therapy. Finally, vitamins may display varying therapeutic effects even in a single disease model. Thus, additional studies are needed in the future. Additionally, there are several treatments that have been investigated less thoroughly in preclinical studies; these are listed in Table 6.^455–462^

**Table 6:** Other alternative therapy for ARDS in pre-clinical study

Medication	Animal model	Main effect	Main effector	Measurement indicator	Potential signaling pathway	Ref
Recombinant antithrombin	LPS	Anti-inflammation Promoting DNA repair	Pulmonary endothelial glycocalyx	IL-1β, IL-6; WGA staining, SEM; Ki-67, PCNA, phosphorylated γ-H2A.X.	None	^455^
Recombinant human thrombomodulin	LPS	Anti-inflammation Promoting DNA repair	Pulmonary endothelial glycocalyx	IL-6, HMGB1; Syndecan-1, WGA staining, SEM; Ki67, HS6ST1 and ESM 1.	None	^456^
Poly-Aspirin	LPS/Bacteria	Reduced lung damage Anti-inflammation Neutrophil phagocytosis	Pulmonary neutrophils	IgM and albumin in BALF; CXCL1, CCL2, TNF and IL-6.	None	^457^
Crocin	LPS	Decreased histopathologic injury Reduced lung permeability Anti-inflammation	Pulmonary endothelial cells	HE staining; FITC-albumin osmosis; SDC-4 and HE staining; MMP-9 expression.	MAPK, HMGB1 and NF-κB pathway	^458^
BAY-1834845	LPS	Anti-inflammation	All cells in lung tissue	HE staining; RNA-Sequencing.	None	^459^
Metformin	LPS	Anti-inflammation Anti-oxidative stress Mitochondrial regulation	Alveolar macrophages BMDMs, PBMCDMs	HE staining; IL-1β; NLRP3 inflammasome activation; Mitochondrial ATP, dynamics.	CMPK2	^460^
Fluorous-Tagged Bioactive Peptides	LPS	Anti-inflammation Anti-oxidative stress Lysosomal membrane stabilization	Pulmonary macrophages	Micro-computed tomography; IL-6, cathepsin B; RNA-Sequencing.	TLRs, NF-κB and MAPK	^461^
β-Glucans	THP-1 macrophages	Anti-inflammation Immunomodulatory activity	Macrophages in vitro	IL-6, IL-8 and TNF-ɑ; IL-10 and IL-22; Phagocytosis.	None	^462^

Some treatments of ARDS show benefits in pre-clinical study, mainly evidenced by anti-inflammatory effect. Their main effectors are cells in lung tissue, including pulmonary endothelial cells, pulmonary neutrophils, and macrophages

*ARDS acute respiratory distress syndrome, *ALI* acute lung injury, *BALF* bronchoalveolar lavage fluid, *BMDMs* bone-marrow-derived macrophages, *CMPK2* cytidine/uridine monophosphate kinase 2, *HMGB1* high mobility group box 1, *IL* interleukin, *MAPK* mitogen-activated protein kinase, *NF-κB* nuclear factor kappa B, *PBMCDMs* human peripheral blood mononuclear cell (PBMC)-derived macrophages, *PCNA* proliferating cell nuclear antigen, *SEM* scanning electron microscopy, *TLRs* toll like receptors, *WGA* wheat germ agglutinin

Interestingly, several novel drug platforms have been reported in recent years. Lung-targeted liposomes are an organ-specific and effective method for drug delivery. The liposomal lipid composition mimics the lung surfactant composition and includes steroids, MPS, and N-acetyl cysteine (NAC), which exert significant therapeutic effects by attenuating pulmonary inflammation.^463^ At the same time, encapsulated dexamethasone decreases oxidative stress-induced lung injury by inhibiting protein accumulation, neutrophil accumulation, and lipid peroxidation in ARDS.^464,465^ Furthermore, liposome-encapsulated trans-crocetin (TC) is longer lasting than free TC, with a longer duration of oxygenation in vitro and an extended half-life in vivo in the context of CARDS.^466^ In addition, larger nanoparticles have greater cellular uptake. These agents are highly adaptable in cells due to the size-dependent anti-inflammatory properties of nanoparticles, resulting in therapeutic effects via activation of anti-inflammatory pathways in ARDS.^467^ In conclusion, these new drug delivery methods are more effective and safer than traditional drugs, providing a new therapeutic direction for the treatment of ARDS.

## Response to treatment

### Different responses to mechanical ventilation

The response of subphenotypes to mechanical ventilation was tested in several clinical trials. Patients randomized to the high- vs. low-PEEP strategy had mortality rates of 24% and 16% for the hypoinflammatory subphenotype, respectively, compared with 42% and 51%, respectively, for the hyperinflammatory subphenotype.^118^ Moreover, focal ARDS and nonfocal ARDS patients respond differently to mechanical ventilation. High PEEP and recruitment maneuvers decrease mortality for patients with nonfocal ARDS, as do high tidal volume, low PEEP, and prone positioning for those with focal ARDS. However, mortality increases substantially when mechanical ventilation parameters are not aligned with the subphenotype.^450^ These results are consistent with Constantin JM’s results, which emphasized the need for different recruitment maneuvers for patients with focal ARDS and nonfocal ARDS.^468^ Moreover, CT-based lung imaging patterns reveal some similarities between the hyperinflammatory subphenotype and nonfocal ARDS, including the mortality rate, incidence of sepsis, and expression of specific lung injury biomarkers, especially plasma sRAGE, which is associated with nonfocal ARDS.^469^ These results suggest that individualized ventilation strategies for patients with ARDS subphenotypes effectively improve patient prognosis. Two ARDS phenotypes were confirmed based on respiratory mechanics, gas exchange, and computed tomography-derived gas and tissue volumes. Compared to recruitable subphenotype, non-recruitable subphenotype is characterized by lower respiratory system elastance, dead space, and total lung tissue, a higher arterial pressure of oxygen/inspiratory fraction of oxygen (PaO_2_/FiO_2_) ratio, a more physiological pH and less inhomogeneous lung. In particular, the non-recruitable subphenotype had a lower success rate of recruitment maneuvers and a higher mortality rate than did recruitable subphenotype.^470^ Additionally, gravity influences the regional distribution of opening and closing pressure, hysteresis and atelectrauma, with higher values in the dorsal lung.^471^ It is likely that the dependent lung is prone to worse physiological conditions. Assessing the lung’s regional behavior with inspiratory and expiratory pressure–volume (PV) curves can help identify ARDS phenotypes and guide personalized mechanical ventilation settings.

### Different responses to fluid therapy

Famous KR et al.’s analysis supported the existence of two ARDS subphenotypes, with higher inflammatory biomarker levels and hypotension characterizing subphenotype 2. In addition, they confirmed that these two ARDS subphenotypes responded differently to a randomized fluid management strategy. Patients with hypoinflammatory subphenotype had 90-day mortality rates of 26% in the fluid-conservative group and 18% in the fluid-liberal group, while these rates were 40% and 50% in these two groups, respectively, in patients with hyperinflammatory subphenotype.^472^

### Different responses to pharmacotherapy

In addition to responding differently to mechanical ventilation and fluid therapy, ARDS subphenotypes also respond differently to pharmacotherapy. Survival was significantly improved in patients treated with simvastatin compared with patients treated with placebo among patients with a hyperinflammatory subphenotype.^147^ However, no treatment effect of rosuvastatin was observed among patients with the hyperinflammatory subphenotype.^473^

## Conclusion

**Fig. 6:**
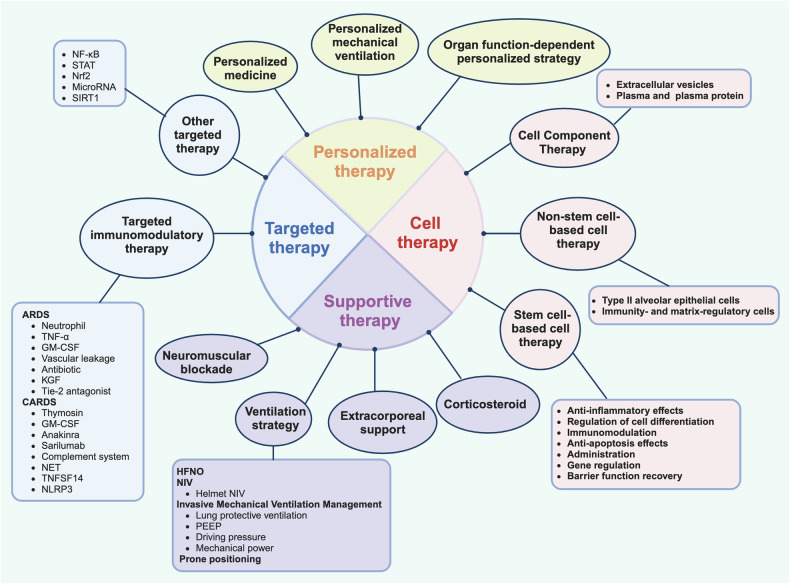
**Main therapies of ARDS**. With the deepening of research, the treatment methods of ARDS have developed many new factions based on traditional treatment methods. Clinical trials have confirmed the effectiveness of cell therapy in the treatment of ARDS, especially stem cells and cell components. In addition, targeted therapy with targeted immunotherapy as its core also shows good therapeutic effects. However, due to the significant heterogeneity of ARDS, emerging evidence has revealed that personalized medicine should be administered in different ARDS subphenotypes. *Targeted therapy: Targeted therapy for ARDS focuses on interrupting or modifying specific molecular, genetic, or cellular mechanisms underlying lung injury and inflammation, thus reducing symptoms and improving outcomes. Personalized therapy: Personalized treatment of ARDS refers to developing personalized treatment methods based on the individual characteristics of the patient, such as genetic makeup, medical history, and unique disease manifestations, to optimize treatment efficacy and minimize side effects. HFNO high-flow nasal cannula oxygen, NIV noninvasive ventilation, PEEP positive end-expiratory pressure, ARDS Acute respiratory distress syndrome, CARDS COVID-19 related acute respiratory distress syndrome, TNF-α tumor necrosis factor alpha, GM-CSF granulocyte-macrophage colony-stimulating factor, KGF keratinocyte Growth Factor, NET neutrophil extracellular trap, TNFSF14 lymphocyte-inducing protein, NLRP3 NOD-, LRR- and pyrin domain-containing 3, NF-κB nuclear factor kappa B, STAT signal transducer and activator of transcription, Nrf2: nuclear factor-E2-related factor 2

In recent years, the incidence of ARDS has been increasing gradually, compounded by the emergence of new causes of viral pneumonia, such as SARS-CoV-2, which make the occurrence and progression of ARDS more complicated. Notably, some novel factors, such as blood product transfusion, e-cigarettes, and ozone, have become risk factors for ARDS, which highlights the need to pay attention to the influence of these factors on ARDS. Over time, substantial progress has been made in understanding the epidemiology and biology of this heterogeneous syndrome. We now know that ARDS is highly heterogeneous in terms of clinical features, causes of lung injury, effective biomarkers, and clinical and biological variables, we also summarized the application of various treatments to treat ARDS in preclinical and clinical practice, shown in Fig. 6. However, personalized medicine approaches for patients with different phenotypes might be a goal of future treatment.

Future directions for ARDS treatment include identifying which treatment approaches apply broadly to any patient meeting the diagnostic criteria and which approaches should be personalized to specific aspects of physiology and biology that could be used to identify a more treatment-responsive subgroup. Traits associated with treatment response can be identified by biomarkers, which align with underlying pathophysiological mechanisms and can be targeted by specific therapeutics or interventions. Alternative biomarkers might also include imaging, physiology and clinical data that reflect an underlying pathophysiological process that could be responsive to therapy. The evidence base for optimal supportive care and interventions in ARDS patients continues to evolve to address areas of uncertainty. As we enter an era of precision medicine for critical illnesses, the future of ARDS management will move towards identifying biological phenotypes and traits associated with treatment response and delivering personalized therapeutic interventions.

To overcome the challenges associated with ARDS treatment, a multidisciplinary approach is required to combine the expertise of clinicians, scientists, and industry partners. This collaboration will facilitate the development and implementation of personalized medicine strategies. Ultimately, the successful implementation of personalized medicine strategies for ARDS treatment will depend on rigorous evaluations of safety and efficacy in large clinical trials, the development of innovative manufacturing and delivery technologies, and the establishment of a sustainable and equitable healthcare system that ensures access to this cutting-edge therapy for all patients in need.
